# Exploring the Regional Diversity of Eukaryotic Phytoplankton in the English Channel by Combining High‐Throughput Approaches

**DOI:** 10.1002/mbo3.70097

**Published:** 2025-11-09

**Authors:** Zéline Hubert, Luis Felipe Artigas, Luen‐Luen Li, Claire Dédécker, Sébastien Monchy

**Affiliations:** ^1^ Université du Littoral Côte d'Opale, CNRS, Univ. Lille, UMR 8187, LOG Laboratoire d'Océanologie et de Géosciences Wimereux France

**Keywords:** Eastern English Channel, eukaryotes, high throughput sequencing, phytoplankton, pulse‐shape‐recording automated flow cytometry

## Abstract

Monitoring marine phytoplankton is essential to understanding marine ecosystems functioning, especially in productive regions like the English Channel. This study applied high‐throughput sequencing (HTS) and automated pulse shape‐recording flow cytometry (PSR FCM) to investigate the spatial and seasonal variability of phytoplankton diversity in French waters of the English Channel during the ECOPEL cruises in April (spring) and July (summer) 2018. Our findings revealed significant seasonal shifts in size, structure, total red fluorescence (FLR, a biomass proxy) and community composition. PSR FCM provided high‐resolution size class discrimination, revealing an increase in picoeukaryote abundance and lower FLR in summer compared to spring. HTS enabled detailed taxonomic insights: in spring, picoeukaryotes (e.g., *Ostreococcus*) dominated in the Western English Channel, except in Finistère/Celtic Seas, where microphytoplankton represented the majority of reads. Nanoeukaryotes (*Phaeocystis*) dominated in the Eastern English Channel. In summer, diversity increased, with co‐dominance of picoeukaryotes (*Micromonas, Bathycoccus*, *Ostreococcus*), microphytoplankton (*Chaetoceros, Leptocylindrus, Guinardia*) and nanoeucaryotes (*Teleaulax*, *Gephyrocapsa*) in the Bay of Seine. Beyond a pronounced west‐east disparity, the Bay of Seine exhibited remarkable taxonomic and functional diversity, with high local contribution to beta diversity (LCBD) values in both seasons. Diversity patterns were strongly influenced by temperature and nutrient concentrations (phosphate, nitrogen), with secondary influences from salinity and turbidity. PSR FCM further revealed sub‐mesoscale variability in abundance and size structure, complementing the mesoscale patterns observed through HTS. This study highlights the importance of integrating both methods to capture fine‐scale phytoplankton dynamics and high‐resolution diversity, thereby enhancing ecosystem management, espcecially in nutrient‐sensitive, productive marine regions. The preprint of the manuscript is available on Authorea (DOI: 10.22541/au.174523338.82769110/v1) and relayed by Archimer (https://archimer.ifremer.fr/doc/00951/106275/).

## Graphical abstract

By combining flow cytometry and high‐throughput sequencing, we reveal seasonal and spatial variations in the size, composition, and diversity of phytoplankton in the English Channel, highlighting the dynamics of pico‐, nano‐, and microphytoplankton and environmental factors, with the Seine Bay as a hotspot of taxonomic and functional diversity.

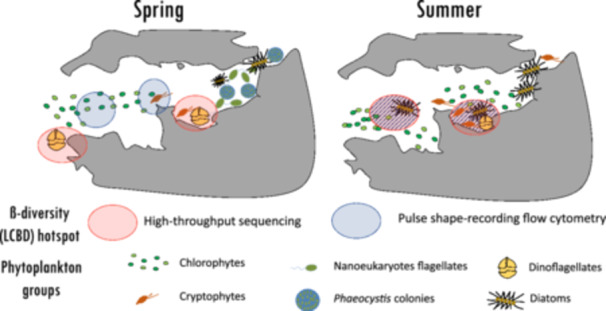

## 1 Introduction

Phytoplankton are responsible for most of the primary production in the ocean. They contribute to the recycling of organic matter by bacterioplankton and facilitate its incorporation into microbial food webs through the microbial loop (Falkowski 2012). Their abundance, composition, functional traits and spatial distribution are influenced by their physical and biogeochemical environment (temperature, salinity, nutrients availability, physical constraints; Edwards et al. 2013; Nock et al. 2016; Di Pane et al. 2022) and by their biotic interactions. Phytoplankton constitute a large polyphyletic group, with their presence in five of the eight eukaryotic “super‐groups” (Simon et al. 2009; Burki et al. 2020). Within these groups, different size classes can be distinguished, with varying effects on the ecology and biogeochemistry of the oceans.

The English Channel (EC) is a marginal sea between the Atlantic Ocean and Celtic Seas and the North Sea and plays an essential hydrological role. The particularity of the EC lies in its complex hydrodynamic regime, characterized by strong tides and currents (Salomon and Breton 1993), which play an essential role in shaping its oceanographic and ecological dynamics. In the Western English Channel (WEC), Atlantic Ocean waters enter the system and flow through central offshore waters towards the North Sea. In addition, the Eastern English Channel (EEC) is subject to significant freshwater inflows to coastal waters, especially in coastal French waters, mainly from the Seine River (Dauvin 2012) and smaller Central and Northeastern estuaries. These riverine inputs are a major source of nutrients such as nitrogen (N), phosphorus (P) and silicate (Si), which are essential for phytoplankton growth and play a key role in supporting primary production and shaping community composition (Cloern et al. 2013). In coastal ecosystems like the EC, natural river discharges combined with anthropogenic inputs create strong nutrient gradients, acting as drivers of phytoplankton distribution and influencing biogeochemical cycles.

Environmental conditions create various ecological niches where phytoplankton communities are structured by nutrient availability, hydrodynamic forcing, but also by their functional traits (Litchman et al. 2012; Karasiewicz et al. 2018; Louchart et al. 2023). Morphological, physiological, and behavioral differences strongly modulate how phytoplankton respond to these environmental gradients, allowing them to exploit resources, interact with other organisms, and contribute to ecological processes such as primary production, nutrient regeneration, and the transfer of matter and energy in the water column (Litchman et al. 2012). Among the most important features explaining phytoplankton ecology, cell size play a central role. It is link to abundance (Marañón 2015), food‐web organization and grazing relationships, growth rate, nutrient utilization, motility and sinking (Sommer et al. 2017), and metabolic rate (Brown et al. 2004). In addition to size, cell morphology and taxonomy further influence ecological functioning. Morphological traits, such as shape and the presence of appendages, affect sinking rates, grazing, motility, and the potential to form blooms, thereby modulating the efficiency of carbon export and energy transfer through the food web (Litchman and Klausmeier 2008; Stanca et al. 2013; Pančić and Kiørboe 2018). It results in different taxa vary in their nutrient requirements, growth strategies, and susceptibility to grazing, which together shape community composition and temporal dynamics in response to environmental gradients, as observed in coastal ecosystems such as the English Channel, influencing both primary production and biogeochemical cycling. Because cell size integrates multiple functional traits, phytoplankton are often categorized into broad size classes: pico‐, nano‐, and microphytoplankton. These groups capture key ecological differences, with each size class displaying characteristic adaptations that influence their contribution to coastal ecosystem functioning. Microphytoplankton eukaryotes, often dominated by diatoms, generally represent the largest part of total phytoplankton biomass in coastal and shelf systems (Leblanc et al. 2012). This compartment has historically been the most studied, mainly through microscopic counts, while total phytoplankton biomass was estimated by chlorophyll *a* concentration. However, in some regions, such as the EEC, nanophytoplankton can account for over 80% of phytoplankton biomass, with significant annual *Phaeocystis globosa* blooms (Breton et al. 2000 2022; Seuront et al. 2006; Grattepanche et al. 2011a; Lefebvre et al. 2011; Hernández‐Fariñas et al. 2014; Genitsaris et al. 2015; Genitsaris et al. 2016). Moreover, eukaryotic picophytoplankton and pico‐cyanobacteria play a major role during seasonal transitions in the English Channel (Tarran and Bruun 2015; Bonato et al. 2016), dominating both photosynthetic biomass and primary production in nutrient‐poor waters such as the open ocean and oligotrophic waters (McQuatters‐Gollop et al. 2024). However, this latter group has often been underestimated due to their small size, which makes it impossible to count, identify and conserve using traditional methods. While picophytoplankton can be quantified using optical techniques such as epifluorescence microscopy and conventional flow cytometry, these methods typically include nanophytoplankton but exclude microphytoplankton, which must be analyzed using inverted microscopy. To obtain a complete and unbiased assessment of phytoplankton on a single approach and without the possible biases of the use of fixatives, automated pulse shape‐recording flow cytometry provides a powerful alternative, enabling the characterization and count of the entire phytoplankton community across a broad size range (0.1–800 µm‐width). This method is based on the recording of morpho‐physiological characteristics such as size, pigment content and physiological state (Dubelaar et al. 1999; Dubelaar and Jonker 2000; Haraguchi et al. 2017; Fragoso et al. 2019) derived from optical features, making it robust enough for addressing a wide range of variables, such as phytoplankton abundance, size, biomass, and processes as cell cycles. However, this quantitative method is ataxonomic and can only be used to define phytoplankton functional groups (PFGs). To fill this gap, high‐throughput sequencing (HTS) of eukaryotes enables finer taxonomic resolution of all phytoplankton size classes, as well as detection of rare species (Nolte et al. 2010). This sequencing is mainly based on the use of 18S ribosomal DNA (rDNA) markers for eukaryotes and 16S rDNA for Bacteria. Stern et al. (2023) have demonstrated the benefits of combining HTS and traditional benchtop FCM during a time series measurements in the Western English Channel, as well as for the study of harmful algae. Therefore, combining PSR FCM with HTS should enable to address, for the first time at least in the English Channel, the whole size range of phytoplankton on both their high spatio/temporal and taxonomical resolution.

This study aims to investigate the spatial distribution, composition and diversity of marine phytoplankton in the sub‐surface waters of the French waters of English Channel during spring and summer, considering both taxonomic composition and functional diversity across meso‐ and sub‐mesoscales. The second objective is to assess the possible environmental and biogeochemical drivers (e.g., temperature, nutrients) influencing these distributions. Finally, the study aims to evaluate the benefit of integrating HTS and pulse shape‐recording flow cytometry methods to characterize phytoplankton diversity and size structure more comprehensively.

## 2 Materials and Methods

### 2.1 Sample Measurements, Collection and Acquisition

The data in this study were collected during the ECOPEL (Pelagic Ecosystems in the EC) cruises, conducted in spring and summer 2018 as part of the French Marine Strategy Framework Directive dedicated cruises for the setting of the Pelagic Habitats Monitoring Programme (French Ministry of Ecology‐CNRS INSU convention). These cruises conducted aboard the Antea R/V (IRD‐French Oceanographic Fleet ‐ FOF), focused on the distribution and dynamics of plankton within the French Exclusive Economy Zone (EEZ) of the English Channel and the southern North Sea (SNS) (Artigas 2018). The cruises took place in two key periods: the onset of the *Phaeocystis globosa* spring bloom and the following summer season, period of possible harmful Algal Blooms in the Bay of Seine. Underway hydrological and PSR FCM measurements were made in vivo along coast‐to‐offshore transects within the French EEZ, from Dunkirk to Brest (Appendix 1). The two legs occurred from April 18 to May 2, 2018 (LEG1, 56 discrete samples for biogeochemical and biological HTS samples) and July 16 to July 31, 2018 (LEG2, 52 discrete samples). These campaigns were analyzed and used within the framework of the Marine Strategy Framework Directive (MSFD) in a complementary way for the seasons not already explored during the optimized fishing campaigns (Baudrier 2017; Jouandet et al. 2020).

#### 2.1.1 Environmental Measures and Sampling

The temperature and salinity of the water column were measured using a CTD probe (SBE19, Sea‐Bird Scientific, USA). In addition, temperature, salinity and turbidity were monitored continuously via the PocketFerrybox system (4H JENA engineering, GmbH), which was connected to the boat's water underway pumping system at—2.5 m‐depth. On the other hand, discrete seawater samples were collected at each station from a depth of 2 meters using an 8‐liter Niskin bottle. Concentrations of nitrite (NO₂⁻), nitrate (NO₃⁻), phosphate (PO₄³⁻), and silicate (Si(OH)₄) were measured in duplicate surface water samples using the Aminot and Kérouel (2004) method with a Futura II autoanalyzer (AMS Alliance, Italy). Ammonium (NH₄⁺) levels were quantified with a fluorometer (Turner Trilogy, Turner Designs Ltd., USA) following the addition of a mixture of sodium sulfite solution, sodium tetraborate decahydrate, and orthophthaldialdehyde (Oriol et al. 2021). Dissolved and POC concentrations were measured using a Shimadzu TOC‐L analyzer (Japan), with samples filtered through GF/F 47 mm filters that were pre‐decarbonated and stored in 20 mL vials acidified with 50 µL of hydrochloric acid. POC and Nitrogen (POC and PON, in μg L⁻¹) were assessed with a NA2100 Frisons CHN analyzer. SPM (in mg L⁻¹) was quantified by the weight difference of pre‐combusted GF/F filters (0.7 µm) before and after filtration. Chlorophyll *a* concentration (Chla, µg L⁻¹) was determined using a Turner 10‐AU fluorometer (10‐AU Field Fluorometer, Turner Designs Ltd., United States) and calculated according to the Lorenzen equations (1967) after acetone extraction following the Holm‐Hansen et al. (1965) method cited in Aminot and Kérouel (2004).

#### 2.1.2 Pulse Shape‐Recording Automated Flow Cytometry Analysis

Phytopankton abundance estimates were based on in vivo samples measured continuously underway from the ship's seawater pump and analyzed directly with a pulse shape‐recording automated flow cytometry (PSR FCM, CytoSense, Cytobuoy b.v., Netherlands). Unlike standard flow cytometers, this instrument is designed for analyzing the full spectrum of phytoplankton community structures, spanning sizes from 1 µm to 800 µm‐width at individual cells/colony level based on their pulse shape profiles (Dubelaar et al. 2004). In addition to counting cells/colonies, this technique addresses cell size (by measuring light scatter) and pigment content/presence by measuring in vivo fluorescence (red, orange and yellow) of each particle. These cytometry signals (Forward Scatter‐FWS as a proxy of size, Sideward Scatter‐SWS as a proxy of granulosity, and Red fluorescence FLR as a proxy of chlorophyll *a* in vivo fluorescence, Orange FLO and Yellow FLY Fluroscence as a proxy of Phycoerythrin‐Phycocyanin in vivo fluorescence) enable the discrimination of phytoplankton into distinct populations, reflecting different functional groups within a sample. This distinction was made manually using cytogram analysis with CytoClus dedicated software (Cytobuoy b.v., Netherlands). Phytoplankton groups were characterized based on their optical and pigment signatures (Bonato et al. 2016; Louchart et al. 2024, Hubert et al. 2025; Robache et al. 2025). The groups were defined and named based on the standardized vocabulary defined by Thyssen et al. 2022. According to this vocabulary, phytoplankton were grouped into functional types based on cell size and pigmentation, following established approaches for flow cytometry‐based community analysis (Dubelaar et al. 1999; Fragoso et al. 2019). This classification captures key ecological traits such as nutrient acquisition strategy, photophysiology, and trophic position, making it particularly relevant for understanding community‐level processes in coastal environments. During the campaign, two automated cytometers were used to ensure uninterrupted sampling in the event of technical issues, as was the case in the Bay of Seine during LEG1 (this detail is documented in the submitted database, Artigas 2018). Moreover, this approach enabled a robust intercomparison between the two instruments, allowing for reliable data completion. Both instruments were calibrated using 1 µm fluorescent reference beads (1.0 μm fluorescent beads, Invitrogen, yellow–green fluorescent) to ensure comparability of optical signals. Data from both instruments were processed using the same gating strategy. Consistency between instruments was verified through parallel sample comparisons, and no significant discrepancy was observed in the parameters of interest.

#### 2.1.3 Environmental DNA

Subsurface seawater samples were collected at each station using Niskin bottle, pre‐screened with a 150 µm mesh to retain larger particles and most metazoan, and then filtered with 0.22 µm Sterivex filters units (Millipore, Burlington, MA, USA) until clogging (volumes going from 142 to 6500 mL) using low filtration pressure peristaltic pump. Samples were immediately stored at −80°C onboard until DNA extraction at the laboratory.

##### 2.1.3.1 Dna Extraction and Sequencing

Total nucleic acids were extracted using Qiagen AllPrep DNA/RNA Mini kit (Qiagen, Hilden, Germany) following the manufacturer's protocol, and DNA concentrations were measured with the Qubit fluorometer (Thermo Fisher Scientific, Waltham, MA, USA). Universal primers 18S‐82F (5'‐GAAACTGCGAATGGCTC‐3', (López‐García et al. 2003) and Euk‐516r (5'‐ACCAGACTTGCCCTCC‐3', (Casamayor et al. 2002) were used to amplify around 480 bp of V2‐V3 regions from eukaryotic 18S rDNA gene. The universal primers S‐D‐Bact‐0341‐b‐S‐17 (5'‐CCTACGGGNGGCWGCAG‐3') and S‐D‐Bact‐0785‐a‐A‐21 (5'‐GACTACHVGGGTATCTAATCC‐3') primers were used to amplify the hypervariable V3‐V4 region of the 16S ribosomal DNA of prokaryotes (Klindworth et al. 2013). The 16S sequences obtained were specifically used to target cyanobacteria, and subsequent analyses focused exclusively on these sequences to study the cyanobacterial component of the phytoplankton community. This approach allowed us to study the overall composition of phytoplankton while ensuring consistency with the diversity observed by automated flow cytometry.

PCR reactions were performed using the Invitrogen Platinum SuperFi DNA Polymerase (Thermo Fisher Scientific, Waltham, MA, USA), products were examined on 1% agarose gel electrophoresis and purified using Agencourt AMPure XP PCR purification system (Beckman Coulter, Brea, CA, USA). A second PCR reaction was used to add Illumina sequencing adapters and sample index. DNA productrs were purified with the Agencourt AMPure XP system, and 18S libraries quality was assessed using Quant‐iT PicoGreenAssay (Thermo Fisher Scientific, Waltham, MA, USA) and Agilent Bioanalyzer 2100 system (Agilent Technologies, Santa Clara, CA, USA). Amplicon libraries from multiple samples were pooled at equal concentrations and the Illumina MiSeq paired‐end sequencing (Reagent Kit v2, 2x250bp) was performed at the sequencing facility of GenoScreen (Lille, France). Sequencing data was submitted to the NCBI sequence read archive database (SRA accession: for 18S PRJNA1242217 and for 16S PRJNA1242094).

##### 2.1.3.2 Bioinformatics and Analysis

The rDNA sequences were processed together using MOTHUR v1.47.0 software following the standard operating procedure (https://mothur.org/wiki/miseq_sop/, last access: 22 March 2025). Briefly, sequences were extracted, demultiplexed, quality filtered and aligned against the SILVA database (http://www.arb-silva.de/, last access: 22 March 2025). Suspected chimeras were removed by using UCHIME software (Edgar 2010) and dereplicated to unique sequences. A total of 7,568,881 rDNA reads (~ 66,000 reads per sample) were grouped into Operational Taxonomic Units (OTUs) at a similarity threshold of 97%, using the mean neighbor method. Singletons, referring to OTUs represented by a single sequence within the entire data set, were excluded, as these are typically indicative of sequencing artefacts. Finally, a total of 7300 OTUs were taxonomically affiliated by using BLASTN against the SILVA v138 database. Sequences were clustered into OTUs at 97% similarity, a commonly applied threshold that provides an intermediate resolution suitable for analyzing phytoplankton community structure and allows comparison with previous studies (Romari and Vaulot 2004; Gilbert et al. 2009, 2012). While amplicon sequence variants (ASVs) can provide finer resolution, recent work has shown that OTUs and ASVs often yield similar ecological patterns (Inglis et al. 2022; Jeske and Gallert 2022), and OTU clustering helps avoid potential over‐splitting of taxa due to natural sequence variability. For phytoplankton‐specific analyses, sequences were classified and analyzed at the genus level, which provides one of the most informative resolutions within phytoplankton communities. Higher taxonomic levels (e.g., lineage) were used only to provide a general overview of eukaryotic diversity.

##### 2.1.3.3 Phytoplankton Size Range Characterization

Based on taxonomic affiliation and physiological information from both cultures and field studies, 4944 OTUs (207,888 reads; 67.7% of total OTUs) were classified as phytoplankton‐organisms capable of photosynthesis, including autotrophs and mixotrophs, as documented in the literature (see Appendix 2). At each station, the 10 major phytoplankton genera, representing between 81.25% and 100% of phytoplankton reads, were manually characterized based on a literature search to assign a size class (pico‐, nano‐, microplankton) and a trophic mode that could indicate potential pigmentation. This analysis allowed for the investigation of the structure of the phytoplankton community and facilitated the comparison with flow cytometry data (Appendix 2). In addition, details on the capacity to form toxic or harmful algal blooms (HABs) were added in appendix 2, based on the continuously enriched list (Lundholm et al. 2025).

### 2.2 Data Analyses

All analyses were performed on R software (R‐project, CRAN) version 4.3.1.

#### 2.2.1 Environmental and Biologic Clustering

Sub‐surface sampling stations were grouped into clusters using Hierarchical Ascending Clustering (Ward) from the Euclidean distance matrix based on environmental parameters. These clusters represent distinct hydrochemical conditions and were subsequently used to define water types (i.e., groups of stations with similar environmental characteristics). Each water type can therefore be interpreted as a distinct ecological setting, facilitating the comparison of biological patterns across different environmental contexts. The same hierarchical clustering approach was also applied to biological datasets. Flow cytometry and sequencing data were clustered using Bray‐Curtis distance matrices, allowing the identification of community‐level structures and their correspondence with environmental water types.

#### 2.2.2 Relationship Between Environmental Parameters and Phytoplankton

A heat map based on Spearman correlations allowed for the visualization of the correlations between environmental parameters. Euclidean distances were calculated for each parameter, and Mantel correlations (with 9999 permutations) were conducted to assess relationships between environmental parameters and phytoplankton community composition determined by HTS and flow cytometry.

#### 2.2.3 Beta Diversity

The beta diversity was addressed using Hierarchical Ascendant Classification (HAC) on square root‐transformed OTUs read numbers or the abundance of Phytoplankton Functional Groups, determined by automated PSR FCM. Clusters were generated based on the Bray‐Curtis dissimilarity matrix (Bray and Curtis 1957), followed by a hierarchical classification using Ward's method (Ward 1963).

Changes in the functional and taxonomic composition of eukaryotic phytoplankton communities were analyzed using the local contribution to beta diversity (LCBD, Legendre and De Cáceres 2013; Rombouts et al. 2019; Louchart et al. 2024). This metric enables the assessment of how unique each site's community composition is in relation to overall beta diversity. A higher LCBD value indicates a site with a distinctive composition, which could reflect the presence of rare species, an unusual species assemblage, or a degraded environment with low species diversity including blooming (potentially harmful) species. Species contribution to beta diversity (SCBD) was calculated to address the species variation between sites. A species with a high SCBD value means that it is heterogeneously distributed between sites and contributes strongly to differences in composition between them. Sites where species with high SCBD values are abundant and dominate the community typically also exhibit high LCBD indices (Legendre and De Cáceres 2013). Both LCBD and SCBD were calculated from PFGs cell/colony and HTS reads abundance data that had been previously Hellinger‐transformed to account for the high variability in species and PFGs abundance along the English Channel and between the two seasons explored. Euclidean distances were computed based on the transformed data, and calculations for LCBD and SCBD were performed using the *beta.div* function from the *adespatial* R package (Dray et al. 2025).

## 3 Results

### 3.1 Environmental Characteristics

**Figure 1:**
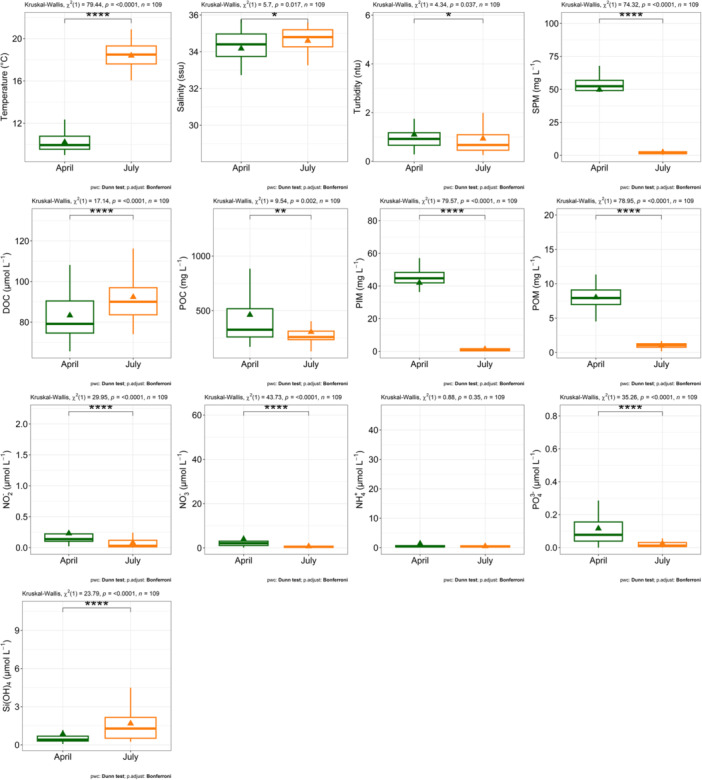
Boxplot of environmental parameters between spring (green) and summer (orange) 2018 ECOPEL cruises in sub‐surface French waters of the English Channel.

A comparison of the abiotic parameters recorded in sub‐surface waters from the two surveys highlights their distinct seasonal characteristics (Figure 1). As anticipated, temperatures were significantly higher in July compared to April. Spring was characterized by statistically lower salinity and elevated concentration of suspended and particulate matter, including suspended particulate matter (SPM), particulate organic carbon (POC), particulate inorganic matter (PIM), and particulate organic matter (POM). Conversely, dissolved organic carbon (DOC) concentrations were higher in summer. Nutrient analysis revealed that waters sampled in April exhibited significantly higher concentrations of nitrite, nitrate, and phosphate, whereas a peak of silicate concentrations was recorded in July.

**Figure 2:**
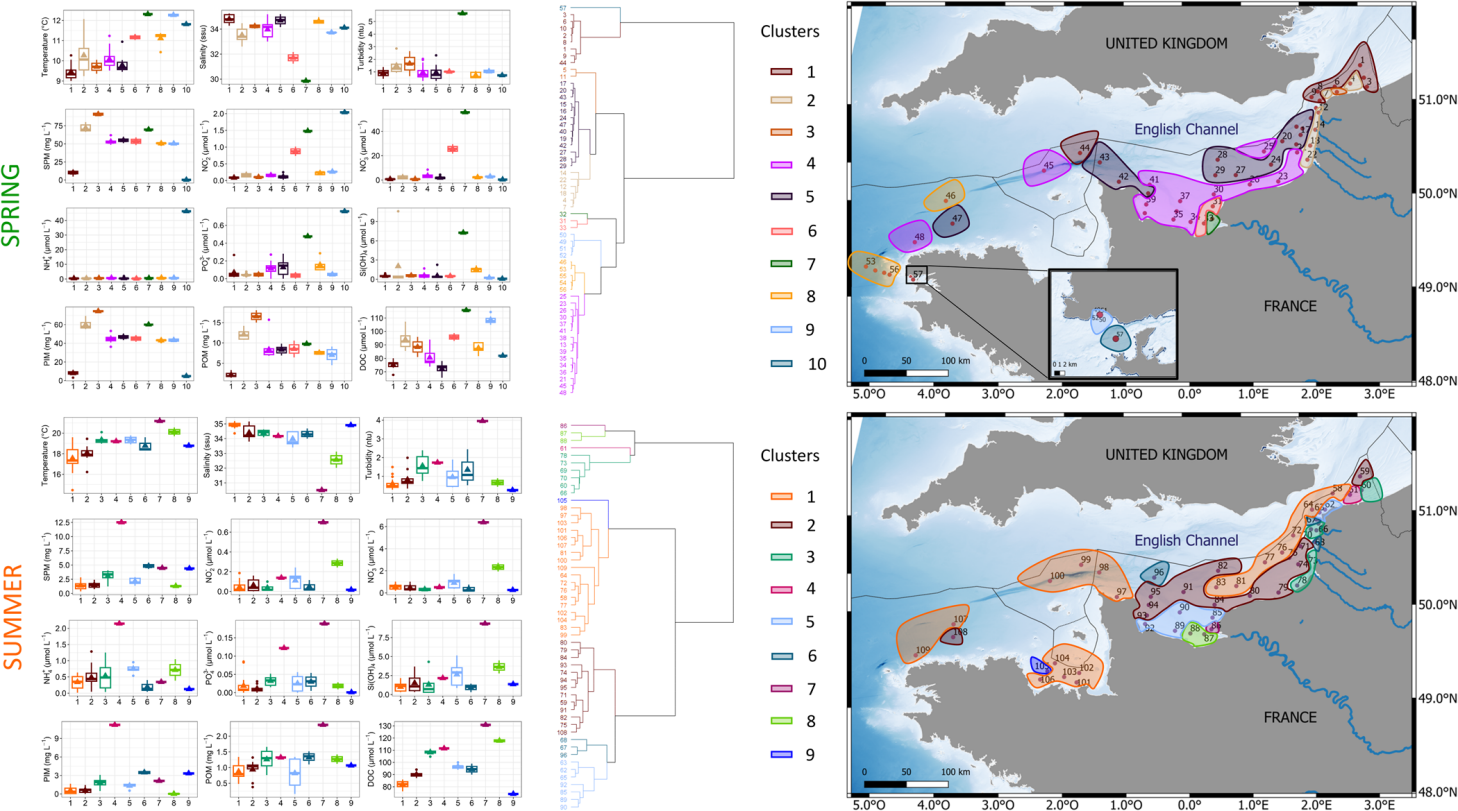
Hierarchical ascending clustering (Ward) from the Euclidean distance matrix of environmental parameters in sub‐surface French waters of the English Channel. Boxplot corresponded to the distribution value of each cluster, represented spatially on the maps to the right. The top panel corresponds to spring and the bottom to summer 2018 ECOPEL cruises.

Given this marked seasonality, Euclidean clustering of environmental data was performed by season to delimit zones with similar environmental characteristics (Figure 2). Regardless of season, a clear environmental gradient was evidenced in the Bay of Seine from the Seine river mouth to northern offshore and western bay waters. Stations near the Seine mouth consistently formed a distinct cluster (cluster 7 in both spring and summer). This cluster represented the area most directly influenced by the Seine River, characterized by the lowest salinity value, the highest turbidity and elevated concentrations of nutrients (highest Nitrate and Silicate levels and high levels of Phosphate and Nitrite in spring, highest Nitrate, Nitrite, Phosphate and Silicate levels in summer). In spring, three distinct water types were characterized in the Bay of Seine, extending northward and eastward along the coast of the EEC in the form of a plume of increasing salinity to the coasts of Haute‐Normandy (clusters 7, 6, and 4). In contrast, four water types were observed in the Bay of Seine during summer (clusters 7, 8, 5, and 2). Further north‐eastward, along the coast, the Somme and smaller estuaries formed its own distinct cluster (cluster 2 in spring and cluster 3 in summer) representing the “coastal flow” (Brylinski et al. 1991) that expanded northwards to the Strait of Dover. During summer, offshore waters of the EEC and most offshore and Gulf Normand Breton stations in the Western English Channel (WEC) were grouped into cluster 1, characterized by the lowest summer temperatures, the highest salinity, and minimal nutrient concentrations, but were more fragmented in spring (clusters 4, 5, 8). Meanwhile, the North Sea formed a separate cluster in spring (cluster 1), but these waters became more fragmented in summer (clusters 2, 3, and 4).

### 3.2 Phytoplankton Functional Diversity

**Figure 3:**
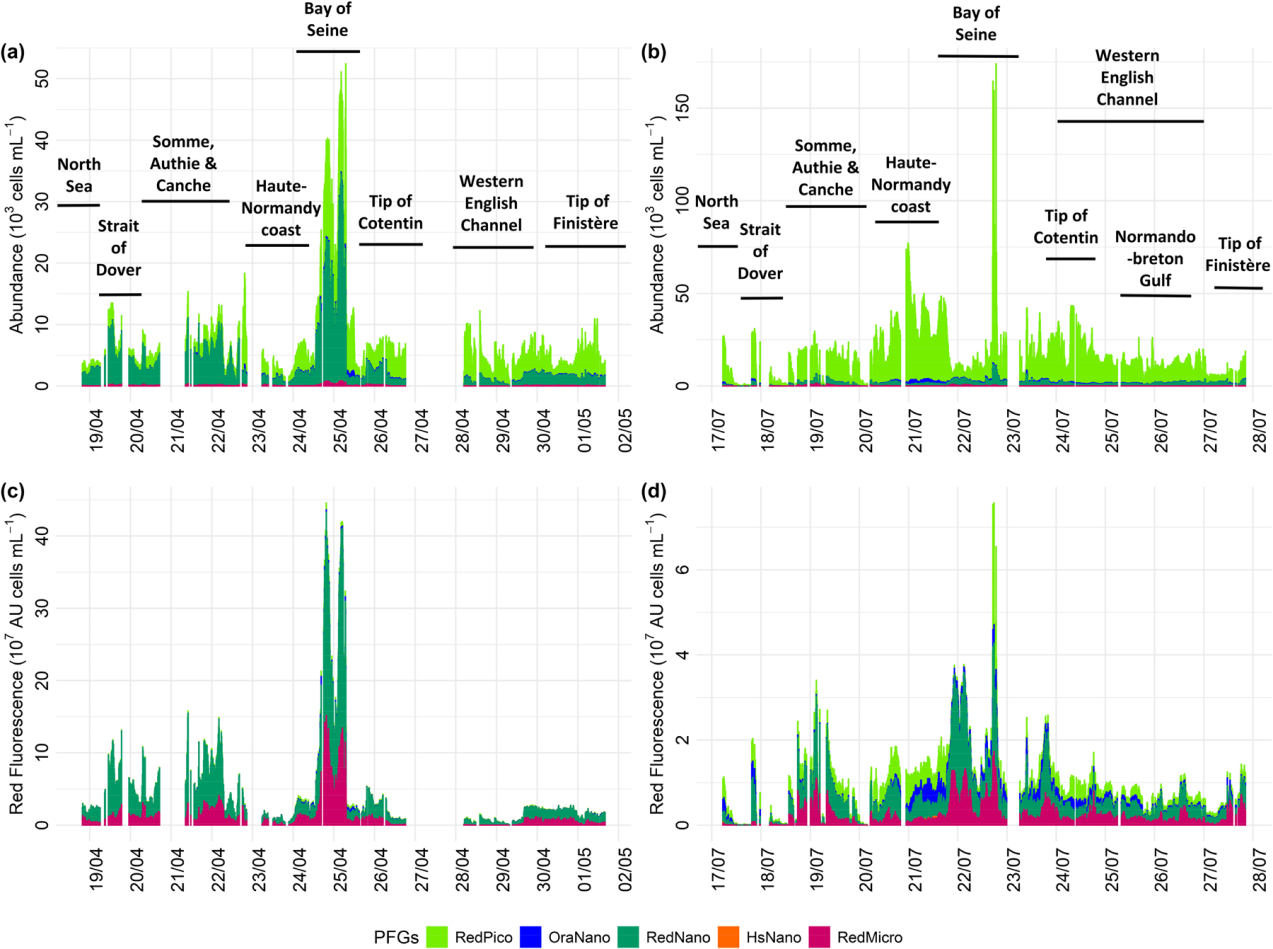
Phytoplankton cells abundance (a, b) and Red Fluorescence (c, d) of eukaryotes phytoplankton functional groups (PFGs) during the spring (a, c) and summer 2018 ECOPEL cruises (b, d) continuous measurements in French waters of the English Channel.

During spring, eukaryotic phytoplankton abundances addressed by automated PSR FCM ranged from 818 to 18,325 cells mL⁻¹, with high values recorded off the estuarine areas (especially in the Bay of Seine; Figure 3a) and the Strait of Dover. The highest phytoplankton abundance characterized the mouth of the Seine estuary, where nanoeukaryote (RedNano) phytoplankton were particularly abundant. Throughout the spring campaign, RedNano dominated phytoplankton abundance in most EEC phytoplankton communities, whereas picoeucaryotes (RedPico) were prevalent in most western (WEC) waters. RedNano was the most dominant group in terms of Red fluorescence (a proxy for chlorophyll *a* concentration and phytoplankton biomass; Figure 3c) across English Channel waters, followed by a notable contribution of microphytoplankton (RedMicro). During the summer campaign, total eukaryote phytoplankton abundances ranged from 161 to 173,972 cells mL⁻¹, with the highest cell density observed in the Bay of Seine. Summer was marked by dominance of RedPico phytoplankton at most stations (Figure 3b). Red fluorescence patterns revealed more diverse contributions compared to spring, with significant contributed FLR of RedNano, RedMicro, cryptophytes (OraNano) and RedPico (Figure 3d). These observations align with chlorophyll *a* concentrations measured at each station (Appendix 3), evidenced that, regardless of the season, the highest chlorophyll *a* concentrations were measured at the Seine River mouth and off the Somme and smaller estuaries along the French coast of the EEC, extending towards the Strait of Dover. However, concentrations were lower in summer compared to spring.

**Figure 4:**
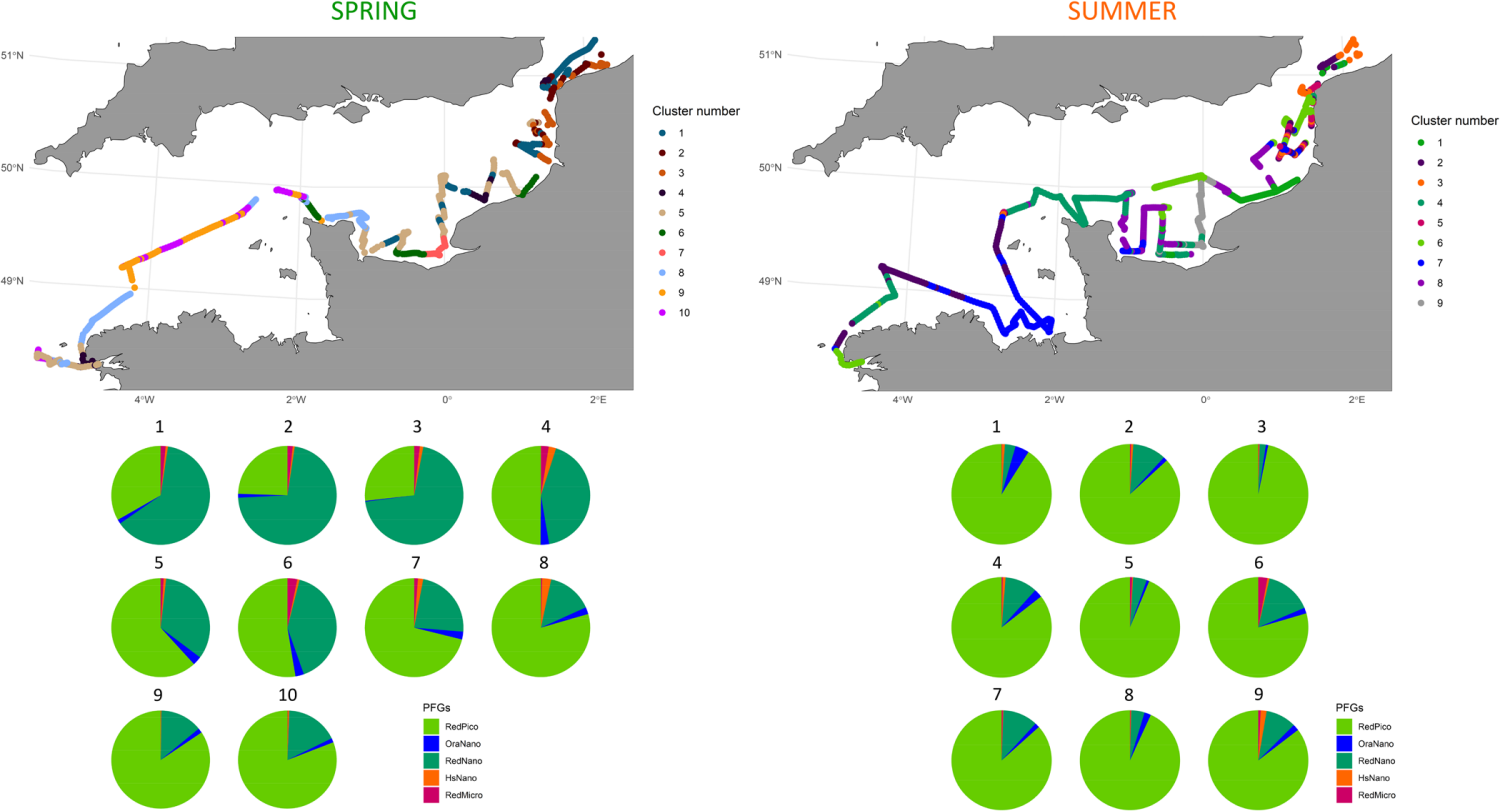
Hierarchical ascending clustering (Ward) from the Bray Curtis distance matrix of cytometric eukaryotes abundance in French sub‐surface waters of the English Channel in spring (left) and summer (right) 2018 (ECOPEL cruises). Pie charts corresponded to the mean value of each cluster, represented spatially on the maps above.

Seasonal spatial clustering of phytoplankton communities defined by Phytoplankton functional Groups (PFGs) revealed differences in phytoplankton communities between English Channel regions and during the two seasons (Figure 4). This clustering showed significant similarities with the clustering of environmental data (Figure 2). The easternmost part of the EEC (North Sea and Strait of Dover) exhibited the highest complexity, combining several clusters in both spring (clusters 1, 2, 3, 4) and summer (clusters 1, 2, 3, 5, 6). This region displayed a complex coastal‐offshore and southwest‐northeast gradient of RedNano (with maximum of 69.89% at coastal cluster 3 in spring and 15% in cluster 6 in summer) and RedPico (maximum of 49.64% at cluster 4 in spring and 96.79% at cluster 3 in summer). In spring, the Bay of Seine was characterized by a community comprising 55.8% of RedNano (cluster 7), together with three other additional clusters (clusters 6, 5, and 1). In contrast, the situation was more complex in summer, with no single dominant cluster characterizing the mouth of Seine estuary, even though cluster 9 was exclusively found in the Bay of Seine. The Bay of Seine was divided into five clusters (clusters 1, 4, 6, 8, and 9) mainly composed of RedPico (79.5% cluster 6% to 93.1% cluster 8), but also contribution of RedNano up to 15% on cluster 6. In spring, clusters 9 and 10 were typical of WEC offshore waters, with the highest contributions of RedPico to the total phytoplankton abundance. Conversely, cluster 8 in spring, showing high proportion of RedPico (80.1%) was indicative of coastal to offshore waters in the western part of the WEC and near the Cotentin Peninsula. Surface waters by the Finistère tip, the Iroise Sea and the Bay of Brest were characterized by clusters 8, 10, 5, 4 of increasing proportion of RedNano (as in some coastal and offshore stations of the Bay of Seine and off Haute Normandy). In summer, clusters 2 and 7 defined coastal to offshore waters of the Gulf Normando Breton in the Western English Channel, characterized by 86% RedPico and about 10% RedNano, whereas off Finistère and Iroise Sea, clusters 4, 2 7 and 6 succeded from off shore to coastal (Bay of Brest) waters (Figure 4).

### 3.3 Eukaryotic Taxonomic Diversity

**Figure 5:**
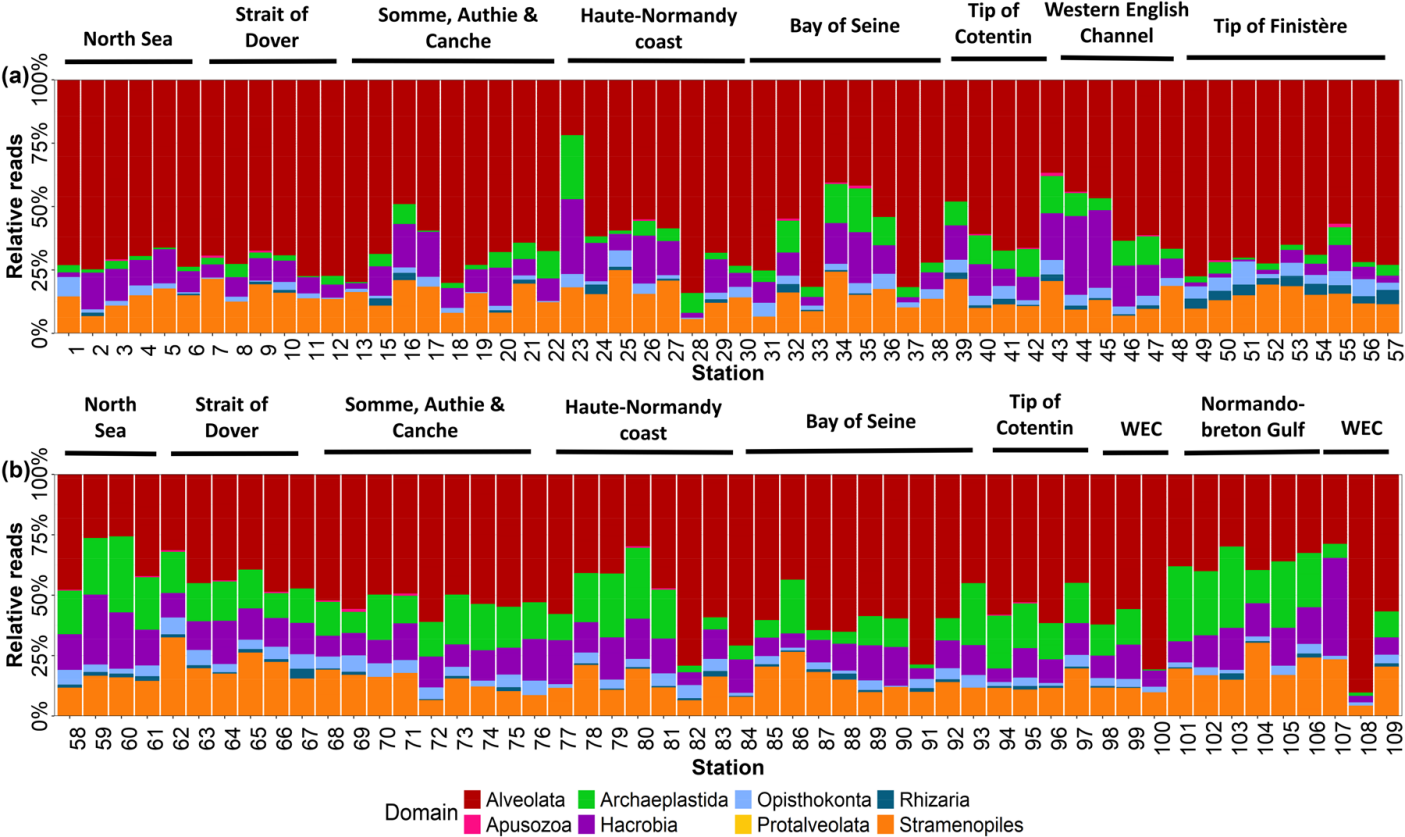
Relative lineagereads of total eukaryotes composition addressed by HTS during the spring (a) and summer 2018 during the ECOPEL cruises (b) in French sub‐surface waters of the English Channel.

Eukaryotic diversity expressed as relative abundance obtained from HTS (Figure 5) highlighted distinct seasonal and spatial variations in community composition in French waters of the English Channel. In spring, the *Alveolata* group was highly dominant (representing about 62% of all reads) except on stations 16 and 23 in the EEC, 34 and 35 in the Bay of Seine and from Cotentin peninsula to offshore WEC waters (43–45). This higher taxonomic group was mainly composed of the lineage *Dinoflagellata* with a contribution of *Ciliophora* from the Bay of Seine to offshore central waters of the WEC. Other significantly present taxonomic group included *Hacrobia* (*Haptophyta, Cryptophyta, Picozoa*) and *Stramenopiles* (*Pseudofungi*, *Sagenista*, and *Ochrophyta*) (Figure 5a). The Bay of Seine exhibited a lineage ‐level composition similar to that of the WEC but showed phylum‐level differences, with significant representation of *Ochrophyta* and *Pseudofungi* in addition to *Chlorophyta*, *Choanoflagellida*, *Haptophyta*, *Cryptophyta*, *Cilliophora* and *Dinoflagellata*. While *Haptophyta* represented the major phylum of *Hacrobia*, the read abundance of *Cryptophyta* and, to a lesser extent, *Picozoa*, increased around Cotentin and the WEC. In the same area, *Archaeplastida* (*Chlorophyta*) were also abundant. *Opisthokonta* (*Mesomycetozoa*, *Fungi and Cercozoa*) were detected at the tip of Finistère and Iroise Sea (stations 49–52).

In summer, a greater and more balanced diversity was observed throughout the English Channel for the dominant groups ‐ *Alveolata, Archaeplastida, Hacrobia*, and *Stramenopiles* (Figure 5b). While *Alveolata* remained the dominant group at some stations like station 108, 100 or 82 where they reached up to 75%, its read abundance decreased by half compared to spring (31% of all reads). This group, primarily composed of *Dinoflagellata* and *Ciliophora*, was distributed across the entire English Channel. In contrast, the proportion of *Stramenopiles*, mainly *Ochrophyta*, increased significantly, particularly in the coastal waters of the EEC, where they accounted for up to 45% of total reads. Their relative reads abundance declined in offshore waters and in the coastal waters of Haute‐Normandy, where *Alveolata*, *Archaeplastida*, and *Hacrobia* replaced them. *Stramenopiles* reached up to 25% in the coastal waters of the Bay of Seine and up to 40% in certain stations of the WEC. *Hacrobia* showed its highest relative abundance in the North Sea, in coastal stations of Haute‐Normandy, in the coastal waters of the Gulf Normand‐Breton, and in offshore waters of the WEC, where they accounted for up to 55% of total reads. The relative contribution of *Hacrobia* to total reads differed between the EEC, where *Cryptophyta* dominated, and the WEC, which exhibited higher diversity due to a greater proportion of *Haptophyta*, as well as contributions from *Picozoa*, *Katablepharidophyta*, and *Telonemia*. *Archaeplastida* reached its highest contribution to total reads in the waters of Normandy and the Gulf Normand‐Breton.

### 3.4 Phytoplankton Taxonomic Diversity Per Size‐Class

**Figure 6:**
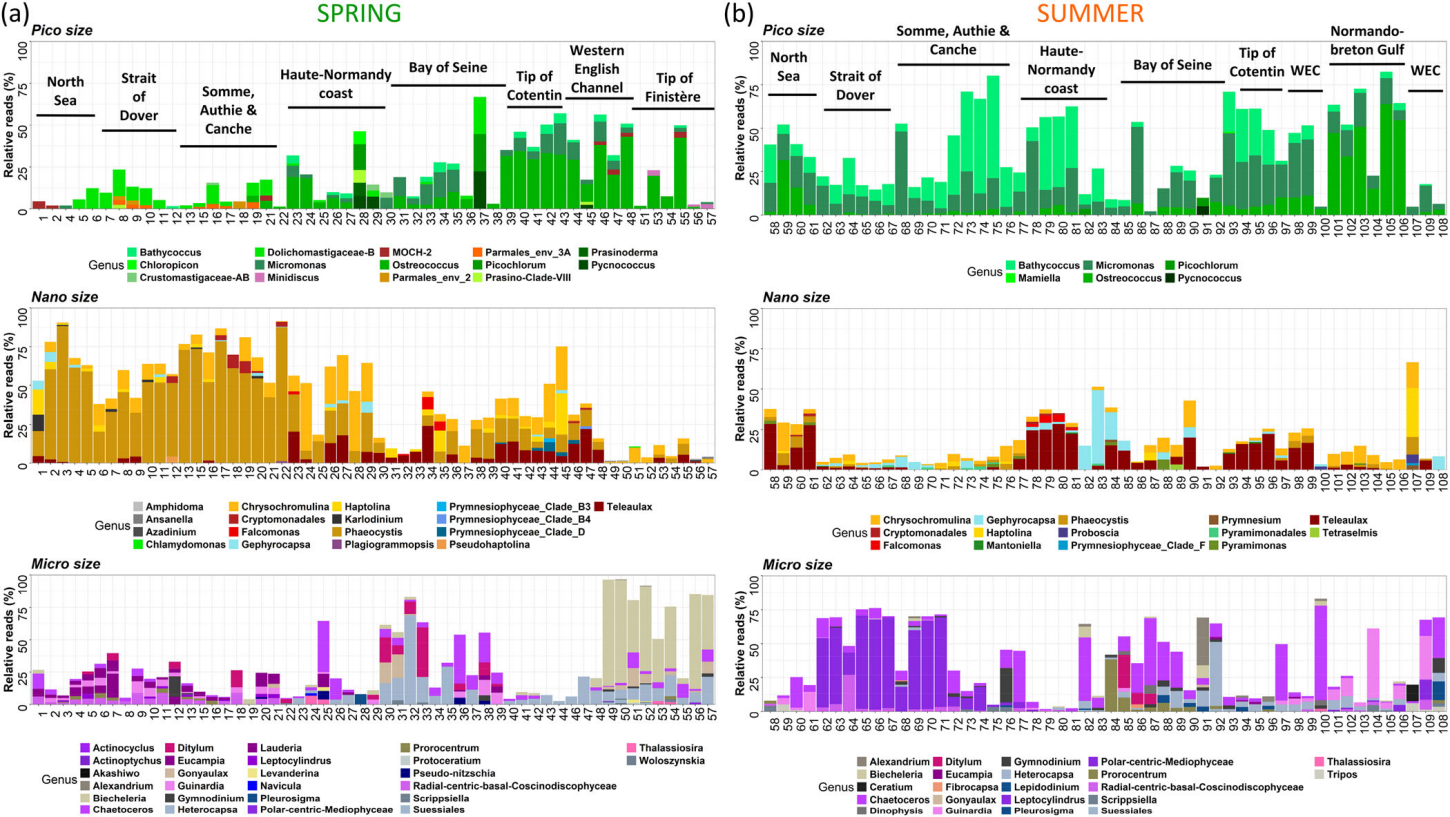
Phytoplankton major genera (10 most abundant per station) in sub‐surface French waters of the English Channel during the spring (a) and summer 2018 ECOPEL cruises (b) grouped by cell size fractions.

The analysis of the ten most abundant pigmented genera per station and during the two seasons offered deeper insights into the composition and distribution of different phytoplankton size classes, defined by HTS in the EC. In spring, pico‐ or microphytoplankton reads dominated in most of the western English Channel, while nanophytoplankton reads were more prevalent in most of the eastern areas (Figure 6a). In terms of composition, the tip of Cotentin (stations 39–44), and certain stations of the Western EC (46, 48, 55) showed a significant contribution of pico‐chlorophyte amongst phytoplankton diversity (ranging between 35% and 56.7% of total reads, represented mainly by the *Ostreococcus* genus). *Micromonas* also contributed to picophytoplankton diversity (12%–15%) in the Bay of Seine stations (35, 34, 33). From the west of the Canche estuary to the Strait of Dover, *Parmales* and *Dolichomastigaceae‐B* accounted for nearly all picophytoplankton diversity. The North Sea stations were characterized by the presence of *Marine OCHrophyte‐2 (MOCH‐2)*. Nanophytoplankton in the EEC, especially from the Bay of Somme to the North Sea, was largely dominated by *Phaeocystis*, reaching over 85% of total reads at stations 3 and 22. In the central and western English Channel (stations 23–48), *Teleaulax* and *Chrysochromulina* contributed more significantly to total reads. Finistère surface waters exhibited a different composition, with the presence of dinoflagellates such as *Ansanella*, *Amphidoma*, and *Azadinium*, along with green algae from the *Chlamydomonas* genus. At the tip of Finistère, the nano‐ocrophyte *Minidiscus* was notably present. The significant presence of dinoflagellates was confirmed by the dominance of microphytoplankton, in particular, the significant contribution of the genera *Biecheleria* and *Heterocapsa* at stations 49–57. From the Bay of Seine (station 39) to the EEC, the proportion of centric diatoms increased, with genera such as *Guinardia*, *Eucampia*, *Ditylum*, *Chaetoceros*, *Lauderia* and *Thalassiosira*. The HTS approach indeed provides added value by detecting potential HAB‐related genera even at low abundance or limited spatial occurrence, for instance, *Prorocentrum* was observed between stations 3 and 16, *Gymnodinium* at station 11, *Heterocapsa* from stations 23–57 (peaking at station 32), *Gonyaulax* appeared at stations 30–31 and 49–57, and *Pseudo‐nitzschia* was detected sporadically at stations 20, 25, 36, and 38.

In summer, the distribution shifted, with smaller phytoplankton alternating its dominance with microphytoplankton, which highest relative contribution to total reads was observed in the Bay of Seine and the EEC, as well as at some stations of the Western English Channel (Figure 6b). The diversity of picophytoplankton at the genus level was lower (less species) than in spring and showed a more distinct distribution. *Ostreococcus* reads remained abundant in the Western English Channel (stations 101 to 106), as observed in spring, but were also abundant in the North Sea waters (stations 59, 60). *Micromonas* and *Bathycoccus* dominated the rest of the English Channel reads, with *Bathycoccus* being particularly prominent between the Bay of Seine and the Bay of Somme. Nanophytoplankton diversity was largely characterized by the strong presence of cryptophytes, particularly *Teleaulax* (from stations 77–99; OraNano on PSR FCM analysis), and coccolithophorids (stations 82–84; HsNano on PSR FCM analysis). *Chrysochromulina* also contributed to nanophytoplankton diversity at several stations. Between the Bay of Somme and the Strait of Dover (stations 62–71), the genus *Leptocylindrus* was highly represented, accounting for up to 68% of total reads. The Bay of Seine, along with stations 97 and 100, exhibited high proportions of the genus *Chaetoceros*. Stations 97 to 109 in the Western English Channel showed the presence of *Guinardia* (representing from 3% to 51% at station 104). A bloom of *Prorocentrum* sp. (38% of reads) characterized station 84 off the Seine estuary, whereas the genus *Alexandrium* represented an important percentage of total reads (35%) at station 91 offshore the Bay of Seine. Other potential HAB‐forming genera such as *Dinophysis*, *Gymnodinium*, *Heterocapsa*, *Lepidodinium*, and *Tripos* were also present.

**Figure 7:**
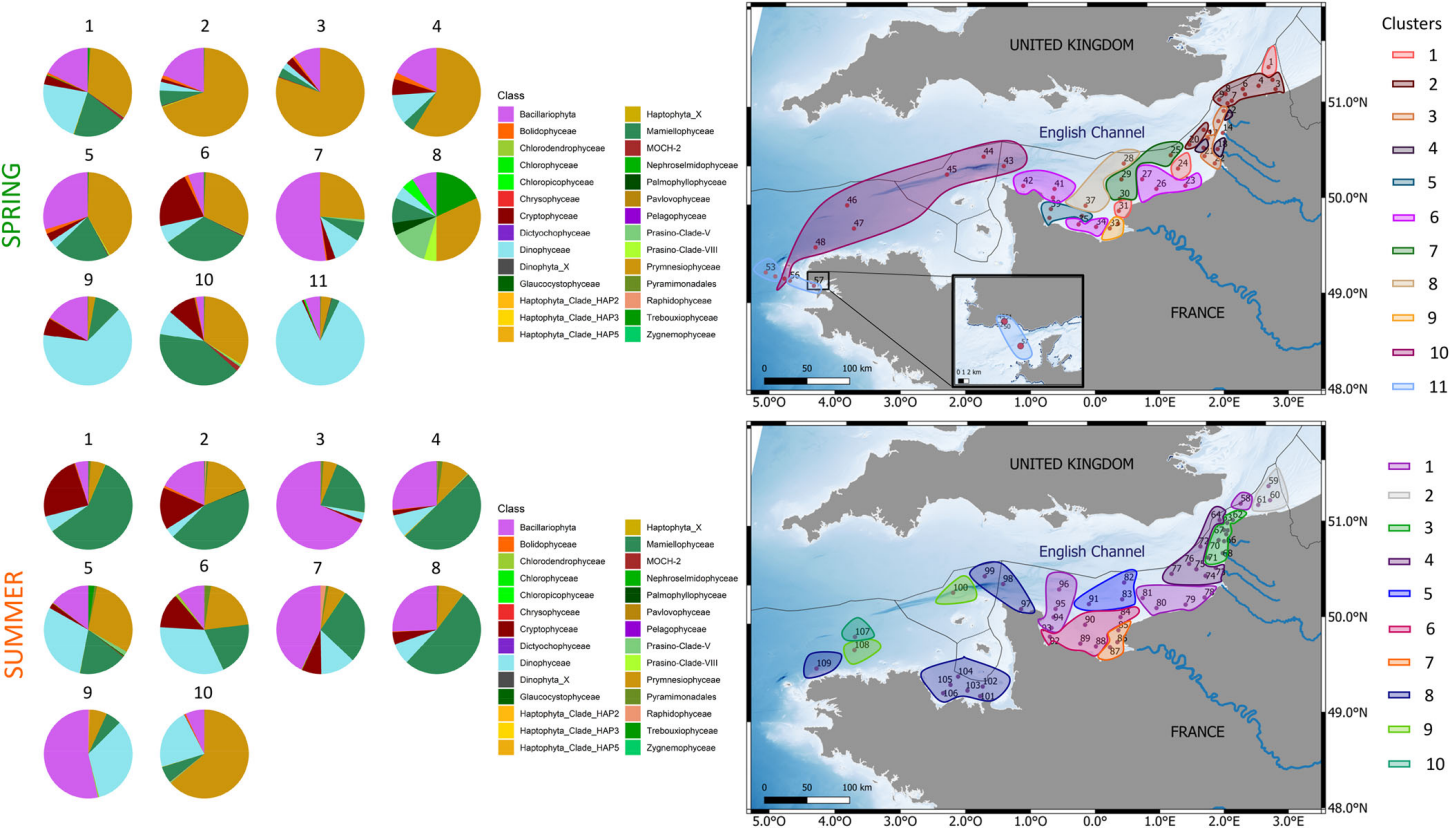
Hierarchical ascending clustering (Ward) from the Bray Curtis distance matrix of taxonomic composition in French sub‐surface waters of the English Channel. Pie charts corresponded to the mean value of each cluster (left) and their spatial location (right). The top panel corresponded to spring and the bottom to summer.

The clustering of phytoplankton communities based on OTUs appears to yield a similar spatial variability compared to that obtained by flow cytometry (Figure 7).

In spring, sub‐surface waters off the Finistère (Iroise Sea) formed a distinct subset (Cluster 11), whose taxonomic composition was largely dominated by *Dinophyceae* exception was for Station 55, which exhibited a composition more similar to the rest of central and offshore waters of the Western English Channel (Cluster 10). Station 55 showed significant contributions from *Mamiellophyceae* (40.81%), *Prymnesiophyceae* (33.47%), and *Cryptophyceae* (10.27%) to the total composition. Coastal waters off Cotentin Peninsula, in the central Bay of Seine and Haute Normandie, constituted a cluster (Cluster 6) characterized by near‐equal contributions to total reads from *Mamiellophyceae* (32.56%) and *Prymnesiophyceae* (31.21%) and the highest contribution of *Cryptophyceae* to the total diversity across all clusters (21.27%). The closest station to the Seine estuary hosted a unique assemblage (Cluster 9) with the lowest contribution of *Prymnesiophyceae* (2.54%), the second‐highest contribution of *Dinophyceae* (64.74%), and 16.18% of *Bacillariophyta* (e.g., *Chaetoceros, Guinardia, Ditylum, Lauderia* or *Thalassiosira*). Cluster 7 represented the Seine's area of influence, extending eastward, with 52% *Bacillariophyta* (e.g. *Chaetoceros, Guinardia, Ditylum, Lauderia or Pseudo‐Nitschia*), 10.20% *Dinophyceae* (e.g. *Levanderina, Heterocapsa* or *Biecheleria*), and 26% *Prymnesiophyceae*. Surface waters off the Bay of Veys (cluster 5), showed a diversity close to that of cluster 4, with 41.2% of *Prymnesiophyceae*, 29.6% of *Bacillariophyta* and 20.3% of *Mamiellophyceae*. In parallel, Cluster 8 (corresponding to Bay of Seine offshore waters) exhibited a more complex composition, including 18.2% *Trebouxiophyceae*, 31.82% *Prymnesiophyceae*, 4.54% *Prasino‐Clade‐VIII*, 13.6% *Prasino‐Clade‐V*, 4.5% *Palmophyllophyceae*, 9.1% *Mamiellophyceae*, 4.55% *Dinophyceae*, 4.55% *Chloropicophyceae*, and 9.1% *Bacillariophyta*. From the Somme estuary to the Strait of Dover, coastal waters were characterized by Clusters 3 and 4, with a high contribution of *Prymnesiophyceae* to total reads (80.39% and 58.8%, respectively). Stations further offshore and into the North Sea, were grouped in Cluster 2 and showed the second‐highest read abundance of *Prymnesiophyceae*. The northernmost station showed a distinct composition that grouped into Cluster 1 with a mixed composition of *Bacillariophyta*, *Dinophyceae*, *Mamiellophyceae* and *Prymnesiophyceae*.

In summer, surface North Sea waters exhibited a unique taxonomic composition with Cluster 2, comprising 17.25% *Bacillariophyta (Guinardia, Chaetoceros)*, 15.29% *Cryptophyceae (Teleaulax and Cryptomonadales)*, 43.3% *Mamiellophyceae (Bathycoccus, Ostreococcus* and *Micromonas)*, and 17.4% *Prymnesiophyceae (Chrysochromulina, Phaeocystis and Haptolina)*. Cluster 1 included a transition station between the Strait of Dover and the North Sea, as well as the coastal transition zone between the Bay of Seine and the Bay of Somme (Haute Normandy), along with a transect north of the Bay of Veys. Coastal waters between the Authie estuary and the Strait of Dover grouped into Cluster 3 (68% *Bacillariophyta*), while offshore waters belonged to Cluster 4. The mouth of Seine stood out with a unique composition with station grouping into Cluster 7, which included 1.92% *Raphidophyceae* (*Fibrocapsa*), absent in other clusters. Clusters 6 and 5, characterized by an increasing proportion of *Prymnesiophyceae* (*Chrysochromulina* and *Haptolina*) and *Dinophyceae* (e.g. *Prorocentrum*, *Alexandrium* or *Gymnodinium*) and decreasing proportion of *Mamiellophyceae*, represented offshore waters of the Bay of Seine. Western English Channel surface waters consisted of three distinct clusters (Clusters 8, 9, and 10), with markedly different average compositions from the EEC. These differences included a strong presence of *Prymnesiophyceae* (60.68%; Cluster 10) and higher contributions of *Bacillariophyta (Chaetoceros, Thalassiosira)* and *Dinophyceae* (*Alexandrium*, *Biecheleria*, *Dinophysis*, *Gymnodinium*, *Lepidodinium*; Cluster 9). These three clusters showed different distributions, with some clusters such as cluster 8 grouping stations from different geographic areas, such as coastal and offshore waters of the Cotentin Peninsula, the Gulf Normando Breton, and off the Norhtern Finistère, whereas cluster 9 was only characterized in offshore WEC waters and cluster 10 by a single offshore WEC station.

### 3.5 Phytoplankton Composition and Environmental Links

In spring, distance correlations and the statistical significance of Mantel's r‐statistic indicated that physicochemical properties, specifically temperature and dissolved inorganic phosphorus (DIP), were strongly correlated with the phytoplankton taxonomic composition (*p*‐value < 0.001; Mantel's r ranging from 0.2 to 0.4). Additionally, dissolved inorganic nitrogen (DIN) and DIP showed strong correlations with the community functional composition identified by flow cytometry (Appendix 4). In summer, the results of Mantel's test showed that turbidity was the only parameter strongly correlated with the functional phytoplankton composition (Appendix 4).

### 3.6 Phytoplankton Diversity Across English Channel and Seasons

#### 3.6.1 Beta Diversity

**Figure 8:**
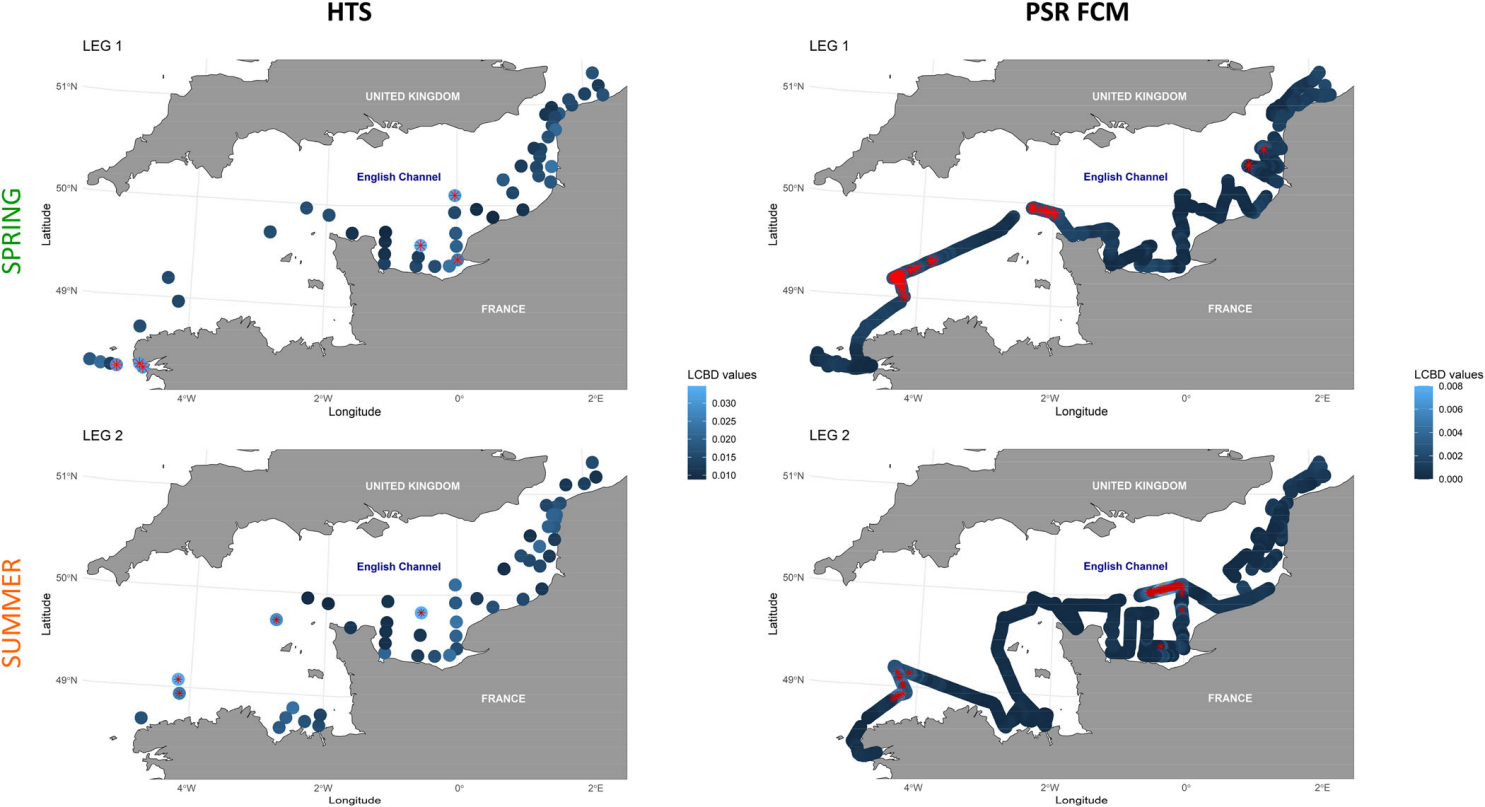
LCBD in sub‐surface waters during spring (top) and summer (bottom) 2018 ECOPEL cruises across the English Channel using HTS and PSR FCM. Red star (inside stations dots) indicates significant LCBD (*p* ≤ 0.05).

The spatial phytoplankton assemblages were analyzed using LCBD values. The highest and most significant values indicated potential spatial shifts in phytoplankton composition, as determined by DNA high‐throuput sequencing (HTS) and automated pulse shape‐recording flow cytometry (PSR FCM, Figure 8). In spring, elevated and statistically significant LCBD values were observed in the Bay of Seine and at the tip of Finistère for HTS, and off the tip of Cotentin and Western English Channel (WEC) offshore waters for flow cytometry. During summer, sub‐surface offshore WEC waters exhibited statistically higher LCBD values for both HTS and flow cytometry. Moreover, some stations in offshore (HTS and PSR FCM) and coastal waters of the Bay of Seine also showed elevated LCBD values.

**Figure 9:**
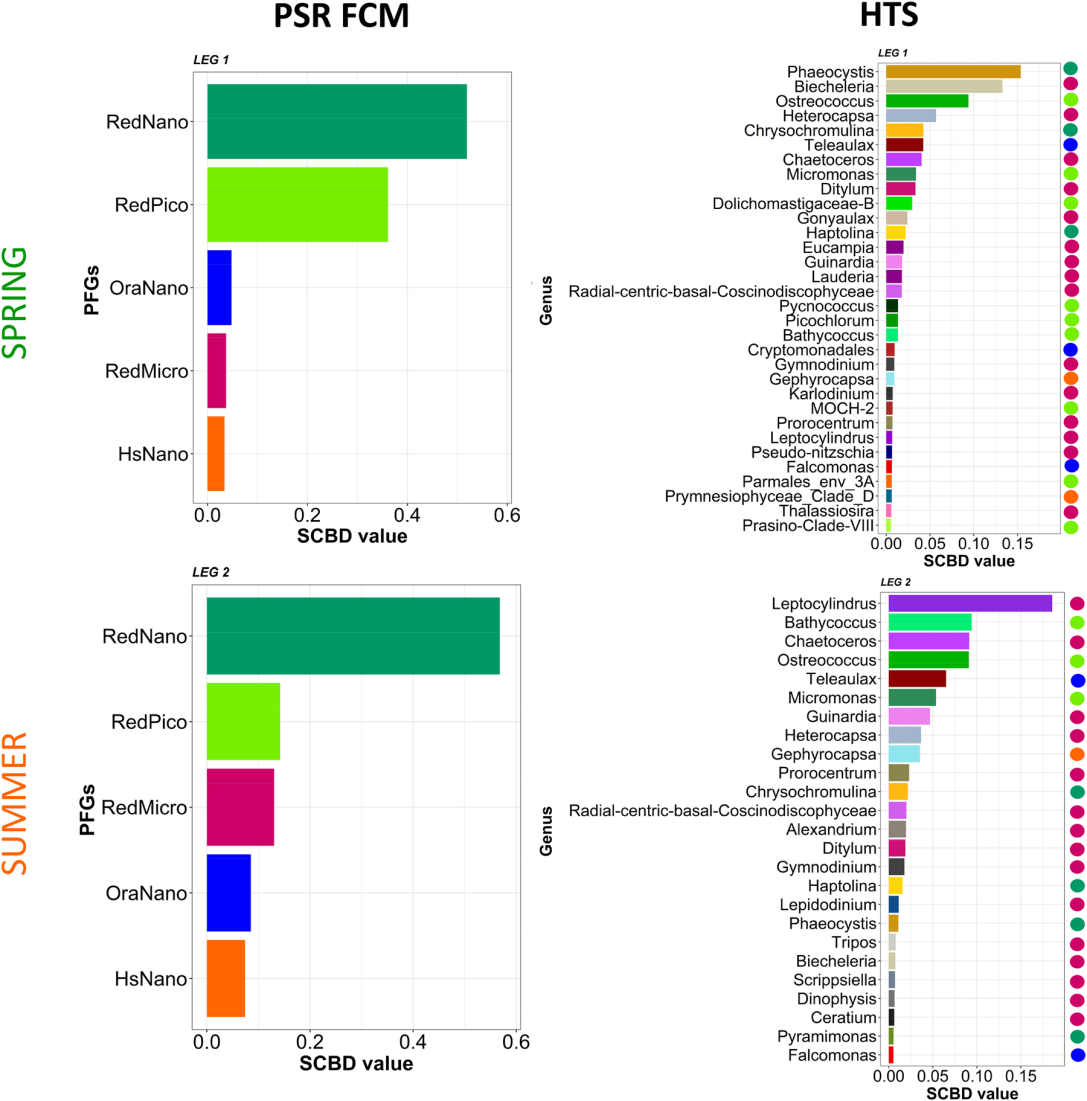
Species contribution to beta diversity (SCBD) of main genera defined by PSR FCM and HTS in spring (top) and summer (bottom) during the 2018 ECOPEL cruises in French waters of the English Channel. Only groups with above the seasonal (spring or summer) SCBD average were showed. Colors dots at the right of HTS plots indicated correspondence with PFGs (dark green: RedNano, light green: RedPico, pink: RedMicro, blue: OraNano, orange: HsNano).

The SCBD values identified the genus (HTS) or PFGs (Phytoplankton Functional Groups; PSR FCM) that contributed most to local beta diversity. In spring, high SCBD values were observed for *Phaeocystis* (associated with RedNano), *Biecheleria* and *Heterocapsa* (both associated with RedMicro), and *Ostreococcus* (associated with RedPico) (Figure 9). Flow cytometry data showed that RedNano and RedPico were the primary contributors to local beta diversity during this leg (Figure 9). In summer, the SCBD highlighted *Leptocylindrus* and *Chaetoceros* (RedMicro), *Bathycoccus, Ostreococcus*, and *Micromonas* (RedPico), as well as *Teleaulax* (OraNano), as key contributors, with RedNano being the dominant marker given by flow cytometry.

### 3.7 Cyanobacteria Diversity and Abundance

**Figure 10:**
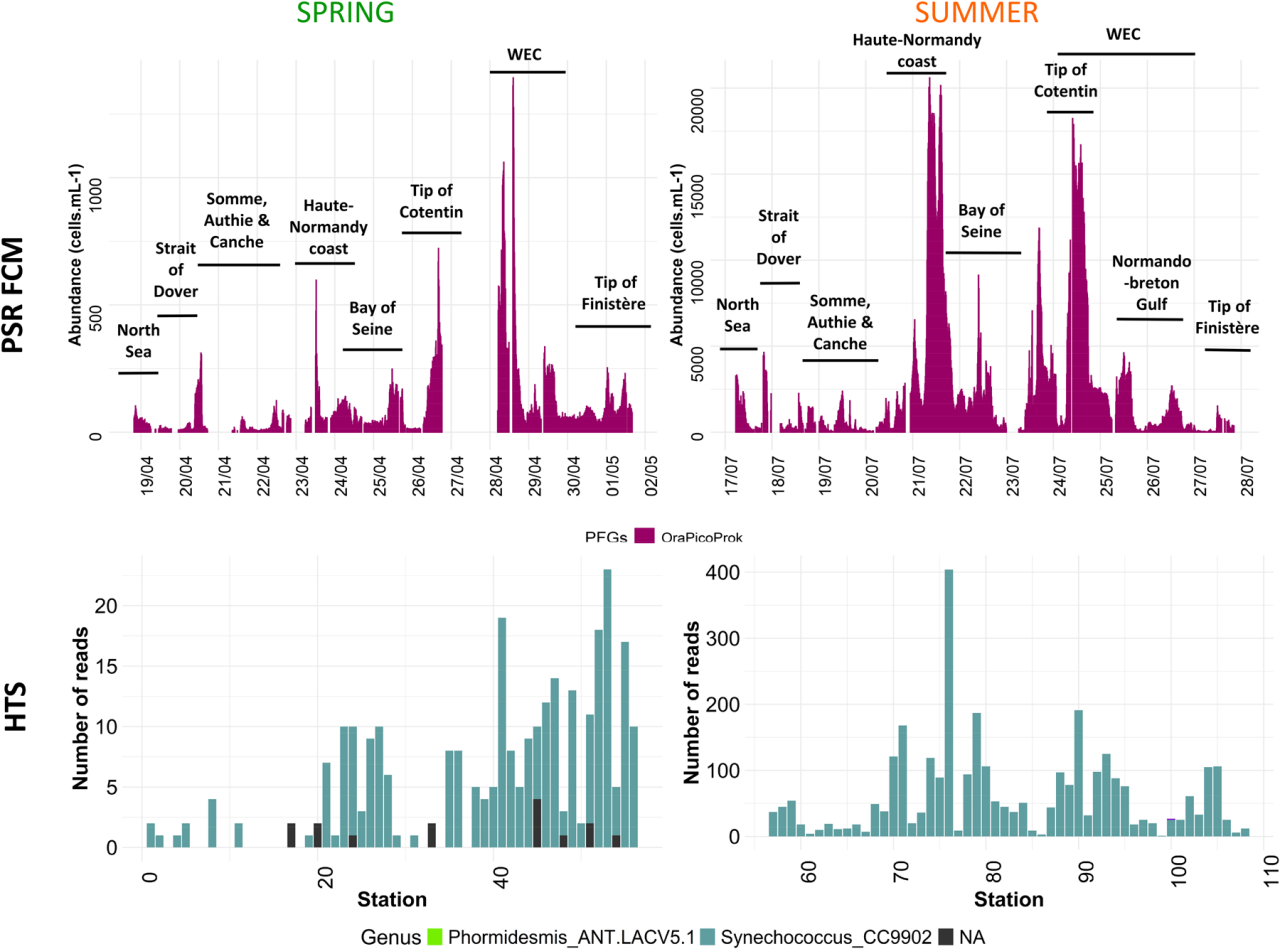
Cells abundance of OraPicoProk phytoplankton functional groups (PFGs) and number of cyanobacteria reads during the spring (left) and summer 2018 ECOPEL cruises (right) continuous measurements in French waters of the English Channel.

In addition to eukaryotic abundances, the photosynthetic prokaryotic component was also analyzed during both spring and summer campaigns. Automated flow cytometry results showed that pico‐Cyanobacteria (OraPicoProk) exhibited a distinct distribution pattern compared to other phytoplankton functional groups (Figure 10). Abundance in summer were higher than in spring. Moreover, during both seasons, their maximum abundance was observed in the Western EC. In summer, high cyanobacterial abundance was also noted in the area between the Seine and Somme estuaries. The photosynthetic cyanobacterial community, analyzed by HTS, revealed three distinct genera: *Phormidesmis ANT.LACV5.1*; *Synechococcus_CC9902* and unidentified taxa (*NAs*, due to limitations in classification/affiliation). *Synechococcus_CC9902* dominated the data set, accounting for nearly all sequencing reads. However, the total number of reads was 20 times higher in summer than in spring, indicating a significant seasonal variation, in agreement with observations made through automated flow cytometry analysis.

## 4 Discussion

The present study reports for the first time the combination of two high‐throughput approaches, discrete (HTS) high taxonomical resolution and continuous high spatio‐temporal resolution (automated PSR FCM), in the French waters of the English Channel, over two seasons.

### 4.1 Environmental Characteristics

Water temperature showed higher variations in the Eastern than in the Western English Channel, reflecting typical seasonal patterns and differences in tidal dissipation and bathymetry, which could lead to a stratification in WEC compared to EEC (Louchart et al. 2020). This combination is known to potentially affect phytoplankton growth rates, metabolic activities and community structure (Regaudie‐de‐Gioux and Duarte 2012; Dauvin 2012; Mousing et al. 2014). Moreover, in addition to seasonal variability, an exceptional warming of the sea surface occurred in the English Channel during the summer of 2018 (Ross Brown et al. 2022). The results of our study seem to indicate that the heatwave had mostly affected coastal areas and the Bay of Seine in July 2018. Salinity levels in the English Channel varied from West to East, with lower values observed off local estuaries, particularly in the EEC, indicating a freshwater influence and the presence of the Seine dilution plume and the EEC “coastal flow” (Brylinski et al. 1991; Dauvin 2012). Surface salinity values of our study were consistent with normal gradients of the English Channel region (range between 26 and 35.5), though variations may occur depending on wind speed and direction as well as rainfall, potentially leading to lower salinity levels due to increased freshwater input (Kelly‐Gerreyn et al. 2006; Louchart et al. 2020; Lefebvre and Devreker 2023). The areas between these river‐influenced regions (ROFIs) also exhibited distinct patterns: in the Bay of Seine, the Orne, Vire, Douve, and Seine estuaries contributed to freshwater and nutrient inputs with strong coastal gradients in both spring and summer (Cugier et al. 2005). Similarly, in the Bay of Somme and the EEC, the “coastal flow” ROFI played a key role in shaping salinity distribution. In contrast, no strong coastal‐offshore gradient was observed in the Gulf Normando‐Breton (not sampled in spring because of very bad weather conditions) or in the WEC, except for the Bay of Brest. This suggests a weaker influence of freshwater inputs in these areas, likely due to distinct hydrodynamic patterns (Ménesguen and Gohin 2006). These hydrodynamic characteristics influenced the water clarity due to the presence of SPM but also phytoplankton cells during blooms, and nutrient concentration, which varies seasonally as stated in the EEC (Lefebvre et al. 2011). In addition, other environmental factors not accounted for in this study, such as photosynthetically active radiation (PAR), bathymetry, and the stability of the water column, may also play a role in shaping the distribution of phytoplankton communities (Louchart et al. 2020). In particular, variations in PAR, especially in coastal region, are known to strongly influence photosynthetic activity, vertical distribution, and seasonal succession of phytoplankton, and may thus represent a key driver of local community dynamics (Gerbersdorf and Schubert 2011; Churilova et al. 2020; McCluskey et al. 2022). In the English Channel, variations in PAR are likely to influence the depth of the euphotic zone and interact with strong tidal mixing and high turbidity, thereby affecting both the photosynthetic activity and the distribution of phytoplankton communities. Besides these physical drivers, nutrient concentrations also play a critical role in shaping phytoplankton distributions. In particular, nitrogen and phosphorus are key regulators of primary productivity, while silicate availability is essential for the growth and dominance of diatoms (Lefebvre and Dezécache 2020). The observed nutrient gradients in the English Channel, largely influenced by riverine inputs and coastal‐offshore dynamics, may therefore explain part of the spatial heterogeneity in phytoplankton communities (Brylinski et al. 1991; Huguet et al. 2024). Seasonal depletion of nutrients during periods of stratification and intense primary production could further modulate community composition; favoring taxa adapted to low‐nutrient conditions. These conditions collectively shaped phytoplankton productivity and community structure, highlighting the interplay between physical, chemical, and biological factors in the English Channel. Moreover, although these campaigns aim to capture some of the seasonal variation in phytoplankton, it is important to remind that phytoplankton can vary on timescales of just a few hours, days, or weeks, especially in a region as exposed to wind and currents as the English Channel.

### 4.2 Taxonomic and Functional Phytoplankton Distribution

Phytoplankton functional groups and genera in the English Channel exhibited both spatial and seasonal variations.

While our approach to size‐class discrimination in metabarcoding differs from the conventional fractionation‐based methods used in other studies, it provides an indirect estimation of size‐structure based on species or genus identity. However, these taxonomic proxies do not always strictly correspond to the real size‐classes measured by PSR FCM, which captures in situ cell size distributions regardless of taxonomy (Dubelaar and Jonker 2000; Thyssen et al. 2015). Interestingly, despite these methodological differences, the observed shifts in phytoplankton distribution and composition along environmental gradients were coherent between both approaches, highlighting the interplay between physical drivers (e.g., stratification, mixing, riverine inputs) and biological responses. In fact, variations in temperature and nutrient concentrations likely influence growth rates and competitive interactions (Cross et al. 2015). Similarly, the relative abundance of different taxonomic groups may modulate community dynamics through grazing pressure or allelopathic interactions. These combined effects help explain why certain groups dominate during specific seasons, highlighting the importance of integrating both abiotic and biotic parameters to understand temporal patterns in phytoplankton communities. Shifts in phytoplankton communities can affect zooplankton populations, alter food availability for higher trophic levels, and affect local fisheries. Moreover, changes in the relative abundance of certain taxa may influence the occurrence of harmful algal blooms, with potential consequences for ecosystem health and human activities.

#### 4.2.1 Picophytoplankton Diversity

In spring 2018, the abundance of picoeukaryotes (RedPico) in the English Channel ranged from 6.8 × 10² to 1.5 × 10⁴ cells mL⁻¹. In contrast, during summer 2018, their abundance ranged from 1.6 × 10² to 1.74 × 10⁵ cells mL⁻¹, with the maximum value being more than 10 times higher than in spring in the Bay of Seine. These abundances are consistent with those reported by Not et al. (2004) and Tarran and Bruun (2015) for the WEC, where eukaryotic picophytoplankton concentrations ranged from 1 × 10³ to 2 × 10⁴ cells mL⁻¹ and maximum abundances were observed in May and June (early summer), ranging from 3 × 10⁴ to 8 × 10⁴ cells mL⁻¹. In the WEC, eukaryotic picophytoplankton were known to be predominantly composed of Chlorophyta (Not et al. 2004; Masquelier et al. 2011), with three main genera present in both seasons: *Ostreococcus*, *Micromonas*, and *Bathycoccus*. Masquelier et al. (2011) also highlighted the dominance of picophytoplankton during the summer period. Off the coast of Roscoff, these three genera were observed throughout the year (mid‐2000 to mid‐2001), allowing for a detailed characterization of their presence and seasonal patterns (Not et al. 2004; Romari and Vaulot 2004; Marie et al. 2010): *Bathycoccus* predominates in February, *Micromonas* in April, and *Ostreococcus* in June and October (Marie et al. 2010). By contrast, a more recent study on metabolites in organic particulate matter found that the genus *Ostreococcus* dominated the phytoplankton community in May, especially under high‐nutrient conditions, notably at station L4 in the western English Channel (Llewellyn et al. 2015).

Including photosynthetic picocyanobacteria in the picophytoplankton diversity reveals different seasonal dynamics in both flow cytometry and HTS data. Flow cytometry showed that Cyanobacteria (OraPicoProk) had distinct distribution patterns, with peak abundances in the WEC during spring and summer, indicating favorable environmental conditions like nutrients and temperature (Napoléon et al. 2014). In summer, higher cyanobacterial concentrations were also observed between the Seine and Somme estuaries, reflecting local environmental influences. HTS identified three cyanobacterial genera: *Phormidesmis ANT.LACV5.1, Synechococcus_CC9902*, and unidentified taxa, with *Synechococcus_CC9902* dominating, suggesting it was the primary contributor to biomass. The observed 20‐fold seasonal variation from spring to summer for both FCM and HTS data likely reflects environmental changes such as temperature, light, and nutrients that favor cyanobacterial growth (Napoléon et al. 2014; Bonato et al. 2016). The presence of unidentified taxa indicates limitations in current classification and calls for further research to better characterize cyanobacterial diversity.

#### 4.2.2 Nanophytoplankton Diversity

In spring, the SNS and the east of the EEC were largely dominated by nanophytoplankton (RedNano, OraNano and HsNano) with abundance ranging from 105 to 10,747 cells mL^−^^1^. This size class is composed almost exclusively of RedNano and the genus *Phaeocystis* (confirmed by HTS), which is consistent with long‐standing observations in the EEC during the spring bloom of *Phaeocystis globosa* (Breton 2000; Lefebvre et al. 2011; Monchy et al. 2012; Genitsaris et al. 2015; Lefebvre and Devreker 2023; Skouroliakou et al. 2022, 2024). *Phaeocystis globosa* can account for up to 80% of total phytoplankton biomass in the EEC (Breton 2000; Schapira et al. 2008). Previous observations using automated flow cytometry revealed that during the spring bloom of *Phaeocystis globosa*, the RedNano group corresponded mainly to this species. Moreover, its life cycle characteristics could be tracked based on clustering resolution, allowing the extraction of sub‐groups within this functional group (Guiselin 2010; Bonato et al. 2015, 2016; Louchart et al. 2024). This genus was also found in summer in smaller proportions in the English Channel, except in the Bay of Seine and its area of influence. *Phaeocystis* maximum of abundance in the WEC was previously described in spring/early summer (Widdicombe et al. 2010; Guilloux et al. 2013; Tarran and Bruun 2015). Correspondence between NanoRed and *Phaeocystis* in the EEC is supported by additional microscopic counts not presented here (Artigas 2018; Jouandet et al. 2020). Other haptophyte like *Chrysochromulina* or *Haptolina* were also detected, as stated by previous observations of nanoeucaryotes in the WEC during the early summer (Tarran and Bruun 2015). Moreover, during spring, the Haute‐Normandy, Bay of Seine and the WEC were characterized by an important contribution of Cryptophytes with the genus *Teleaulax* and *Falcomonas* (Seine estuary). They can also be distinguished using automated flow cytometry based on its orange fluorescence linked to phycoerythrin pigment (Li and Dickie 2001), called OraNano according to common vocabulary (Thyssen et al. 2022). *Teleaulax* contributed largely to total reads (28%, station 80 in Haute‐Normandy coast) over all French waters of the English Channel in summer. These two genera were previously described in spring in the Bay of Veys linked with local estuarine influence (important brackish flow from two local estuaries (Douve and Vire; Bazin et al. 2014) and at the SOMLIT‐Astan station (WEC) coastal waters, as one of the dominant taxa (Caracciolo et al. 2022). Cryptophytes were most abundant in late summer and autumn in the WEC, reaching maximum abundances of 1500–2000 cell mL^−^^1^ (Tarran and Bruun 2015), which is consistent with the peak abundance of 2529 cell mL^−1^ for OraNano counted in the present study. OraNano bloomed in the EEC during the *P. globosa* bloom (spring) and in summer (Bonato et al. 2016). Their phycoerythrin and phycocyanin pigments enable them to effectively tolerate varying light conditions (Tarran and Bruun 2015). Additionally, Cryptophytes can acquire nutrients at low concentrations (Skouroliakou et al. 2022) and exhibit mixotrophy, consuming *Synechococcus* (Rammel et al. 2024). In summer, offshore Eastern Channel stations were marked by the presence of coccolithophorids (*Gephyrocapsa*). Flow cytometry detected HsNano primarily in the EEC during summer, especially in the Bay of Seine and along the Haute‐Normandy coast. In spring, they were concentrated around the same area as well as in the offshore WEC waters. This phenomenon was previously described in the WEC, with coccolithophore blooms from June (Garcia‐Soto and Pingree 2009) and high abundance in the EEC in early April and early July (Bonato et al. 2016). Their tolerance to high irradiance, lower nutrient needs, and ability to use organic nitrogen or phosphorus allow coccolithophores to thrive in both high‐nutrient, well‐mixed and low‐nutrient, stratified conditions (Van Oostende et al. 2012).

#### 4.2.3 Microphytoplankton Diversity

Microphytoplankton is known to contribute the most to total phytoplankton carbon biomass (addressed by inverted microscopy), except during the spring *Phaeocystis* bloom in the EEC (Schapira et al. 2008), in spite of the fact that big colonies can represent an important part of total biomass. Consistent with the red fluorescence measured during both campaigns, phytoplankton biomass appears to be ten times higher in spring than in summer, twice in term of chorophyll *a*. Moreover, while in spring almost all of the red fluorescence originates from RedNano and RedMicro populations, in summer, RedPico and OraNano also contribute significantly to the total phytoplankton biomass. The abundance of RedMicro ranged from undetected to 831 cells mL^−1^ in spring with maximum abundance and red fluorescence in the Bay of Seine. In spring, the contribution of microphytoplankton to the total phytoplankton diversity and functional composition is lower in the SNS and the eastern part of the EEC (the same area where the maximum amount of nanophytoplankton and, more specifically, of the *Phaeocystis* genus was observed). In the EEC, different genera of centric diatoms co‐existed in our study (*Chaetoceros, Eucampia, Radial‐centric‐basal‐Coscinodscophyceae, Guinardia, Lauderia, Leptocylindrus, and Ditylum*). Some of these species were described as transient diatom blooms, characteristic of the end of *Phaeocystis globosa* bloom, such as *Chaetoceros socialis* or *Leptocylindrus danicus*, while *Guinardia striata*, *Coscinodiscus* spp. or *Ditylum brightwellii* are more characteristic of winter diatom communities in EEC (Skouroliakou et al. 2022, 2024). This is consistent with the summer phytoplankton diversity, where the *Leptocyindrus* genus reached over 50% in the EEC, well after the spring bloom of *Phaeocystis*. Similarly, in the EEC, the abundance of RedMicro reached its maximum at 1500 cells mL^−^¹ in the Somme, Authie, and Canche estuaries. In summer, in the French waters of the WEC, high read numbers of the *Guinardia* genus were observed, which is similar to previous descriptions offshore Roscoff in late/early summer (Romari and Vaulot 2004). The Bay of Seine and certain stations off the WEC also showed a high number of *Chaetoceros* reads, and this genus has already been documented in the EEC during summer (Jouenne et al. 2007).

In spring, French waters of the WEC and the Bay of Seine were characterized by a large proportion of dinoflagellates such as *Heterocapsa* and *Biechelaria*, although the diatom *Ditylum* makes a major contribution to the phytoplankton community of the Bay of Seine and *Chaetoceros* to the Bay of Veys. The Bay of Seine is known for its strong eutrophication (Passy et al. 2016) and the occurrence of diatom blooms in spring and summer (Thorel et al. 2017). These blooms are followed by frequent proliferations of dinoflagellates, some of which, like those in the *Heterocapsa* genus, can produce toxins (Napoléon et al. 2014; Belin et al. 2021). In addition to this genus, 25 other potentially harmful algal taxa were identified across the two seasonal cruises of the present study (including mainly dinoflagellates and some diatoms). In the rest of the WEC, dinoflagellates are mainly observed in summer (and autumn) (Widdicombe et al. 2010; Napoléon et al. 2013), but this was not the case in the present study. On the other hand, dinoflagellates (including heterotrophic ones) are known to be less abundant in the EEC, ranging between 0.8 × 103 and 40.4 × 103 cell L^−1^, representing only 1%–11% of total phytoplankton abundance (Schapira et al. 2008). Most of these two large phytoplankton groups (diatoms and dinoflagellates) were gathered together through automated flow cytometry and clustered in the RedMicro group (excepted small diatoms like *Minidiscus* or small dinoflagellates). Microscopic counts carried out in parallel and not presented here (Artigas 2018; Jouandet et al. 2020), showed that the contribution of dinoflagellates was greatly overestimated by HTS. These discrepancies between the two approaches have already been highlighted in studies in the North Sea (Käse et al. 2020) and in estuarine areas (Abad et al. 2016), with biases introduced during DNA extraction, gene copy number or genome size in eukaryotes as probable explanations for these differences (Prokopowich et al. 2003; Lin 2011; Martin et al. 2022).

Our results suggest that certain functional groups detected by PSR FCM could correspond to taxonomic groups identified by HTS. Although these links are promising for understanding community structure, they remain hypothetical and will need to be confirmed by experimental validation.

### 4.3 Relations between Phytoplankton Composition and Environmental Parameters

In spring, temperature, salinity, DIN and dissolved inorganic phosphate (DIP) were identified as significant explanatory factors likely to have influenced HTS‐defined genus distribution, while DIP and DIN influenced those from FCM‐defined PFGs. Previous research in the region indicates that *Phaeocystis globosa*, the dominant species in EEC in spring, begins to bloom only when silicate concentrations fall below a specific threshold (Karasiewicz et al. 2018). Furthermore, this genus is well‐adapted to low levels of DIP and nitrogen, leveraging dissolved organic phosphorus and nitrogen for growth (Sanderson et al. 2008; Breton et al. 2017). Previous studies have also noted the preference of *Phaeocystis* for high salinity conditions, which are associated with minimal precipitation and lower turbidity (Karasiewicz et al. 2018). These environmental factors help explain the spatial distribution observed in spring 2018, characterized by a predominant presence of *Phaeocystis* from the Bay of Somme to the North Sea. This pattern may be influenced by the lower turbidity and lower DIP availability in these areas, despite still high nitrogen inputs from ‘coastal flow', which together favor *Phaeocystis* blooms. In contrast, in the Bay of Seine, the consistently high silicate inputs and strong turbidity may limit its proliferation. In summer, only turbidity appears to have significantly influenced eukaryotic PFGs' distribution in our study.

### 4.4 Analyses of Beta Diversity

Coastal ecosystems are known for their strong spatial and temporal heterogeneity, which can influence phytoplankton concentration and composition (Martin et al. 2005). This heterogeneity is closely linked to environmental variations such as temperature (Righetti et al. 2019), salinity (Muylaert et al. 2009), nutrient concentrations (Marañón et al. 2012), light availability, and turbulence (Seuront 2005), as well as biotic interactions like competition (Pal et al. 2009), grazing, or lysis (Grattepanche et al. 2011a, 2011b). High values of LCBD indicate a unique phytoplankton composition at certain sampling stations or measurements compared to the other sites, with a pattern that remains relatively consistent across different methods. However, some differences were observed, particularly in spring and in some offshore areas, likely due to the inherent differences between the two approaches, which target distinct aspects of diversity and operate at different spatial and either on functional or taxonomic resolutions. Louchart et al. (2024) observed that high LCBD values in spring frequently occur under conditions of low salinity and high temperature, which corresponds to the conditions observed in the Bay of Seine, for example. In these environments, the unique community composition is notably influenced by the co‐dominance of RedNano III with RedPico I, RedPico II, and RedNano II PFGs in the water bodies in spring (Louchart et al. 2024). This aligns with our study results, which show that in spring, SCBD values are higher for RedNano and RedPico, highlighting their significant contribution to community differentiation. Species that play key roles in differentiating communities between sites vary between spring and summer. The SCBD depends, in part, on the abundance of species or phytoplankton functional groups (PFGs; Heino and Grönroos 2017; da Silva et al. 2018; Louchart et al. 2024). Automated flow cytometry provides optical traits that are closely linked to functional characteristics (Fragoso et al. 2019), allowing SCBD to be assessed at a higher frequency across the size spectrum of community‐forming phytoplankton. However, functional traits do not directly influence SCBD (Heino and Grönroos 2017; da Silva et al. 2018) but instead affect it indirectly through species' ecological niche characteristics (Wang et al. 2024). These functional traits have a strong influence on phytoplankton community structure (Litchman et al. 2010), and probably we need to explore further FCM‐defined PFGs, in particular by defining sub‐groups (as carried out by Louchart et al. 2020). Future work should improve cluster resolution beyond the current five identified in the English Channel, by both manual exploration, correspondence to images taking (imaging in flow integrated to FCM, correspondence to isolated/cultivated key taxa and lifeforms, and/or through unsupervised automated classification. Linking these results with physiological data (e.g., Fast Repetition Rate fluorometry or LabSTAF—Single Turnover Active Fluorometry) could help better connect clusters to phytoplankton functional traits.

The SCBD applied to HTS method identified *Phaeocystis*, *Biecheleria*, *Ostreococcus*, and *Heterocapsa* as the main genera contributing to site differentiation during the summer season, while *Leptocylindrus*, *Bathycoccus*, *Chaetoceros*, and *Ostreococcus* showed the highest SCBD values in summer. These genera played a crucial role in the composition of phytoplankton communities, significantly contributed to the total diversity at each site but were not necessarily the most abundant. In contrast, rare species contributed less to SCBD than more common species (Heino and Grönroos 2017; Louchart et al. 2024). This was the case for most of the potential HAB‐forming taxa identified in this study. Although a range of potentially harmful algae was detected, the majority were present at low relative abundances, and no significant HAB events were observed during the two campaigns, except for *Phaeocystis*, which was well represented and thoroughly described. This may indicate that their respective blooming conditions were not met during the sampling periods, or that such events were highly localized and therefore not captured by the discrete sampling resolution. However, the SCBD and LCBD are interesting tools for improving our understanding of community ecology, bioassessment and conservation (Legendre and De Cáceres 2013; da Silva et al. 2018; Rombouts et al. 2019) and can be applied to both taxa and functional groups.

## 5 Conclusions

By combining two high‐throughput approaches, we leveraged their complementary strengths—such as high‐frequency continuous measurements for fine spatial resolution, a functional trait perspective, high taxonomic resolution, the ability to detect rare or hard‐to‐culture organisms, and coverage of a broad size spectrum—while mitigating their respective limitations. By combining these two high‐throughput methods, this integrative approach enhances our ability to infer the small‐scale (high spatio‐temporal resolution) distribution of phytoplankton diversity in the English Channel, both from a functional (PSR FCM) and taxonomic (HTS) approach. However, further advances, such as the deployment of autonomous filtration/extraction systems like the environmental sample processor (ESP; Hendricks et al. 2023) or the Robotic Cartridge Sampling Instrument (RoCSI; Tang et al. 2020), could provide complementary insights and improve continuous, in situ monitoring of phytoplankton community dynamics in autonomous platforms at high frequency. The identification of areas with exceptional biodiversity and/or the presence of species potentially harmful to humans emphasizes the importance of these findings for managing coastal waters and maintaining marine water quality of the EC. Furthermore, identifying factors that influence these communities will shed light on the potential impacts of climate change and human activities on marine biodiversity and ecosystem functioning (including the provision of living resources) in the region. While this study focused on two seasons, extending research to autumn and winter in the same area could yield further insights into the seasonal dynamics of phytoplankton communities. Additionally, long‐term monitoring of these two approaches would provide a deeper understanding of the ecosystems' evolution over time.

## Author Contributions

**Zéline Hubert:** conceptualization (lead), writing – original draft (lead), formal analysis (lead), writing – review and editing (equal). **Luis Felipe Artigas:** conceptualization (supporting), funding acquisition and project and analysis coordination (lead), writing – original draft (supporting), writing – review and editing (equal). **Sébastien Monchy:** conceptualization (supporting), formal analysis (supporting), writing – original draft (supporting), writing – review and editing (equal). **Claire Dédécker:** performance of in vivo FCM measurements (lead), treatment of FCM raw data (lead), writing – review and editing (equal). **Luen‐Luen Li:** writing – original draft (supporting), writing – review and editing (equal).

## Ethics Statement

The authors have nothing to report.

## Conflicts of Interest

The authors declare no conflicts of interest.

## Data Availability

Sequencing data have been submitted to the NCBI sequence read archive database (SRA accession: PRJNA1242217). Flow cytometry and environmental data is in the process to be uploaded to the already existing SISMER repository of French Oceanographic Cruises and, precisely, to the ECOPEL 2018 cruises (ARTIGAS Luis Felipe (2018) ECOPEL 2018 cruise, RV Antea, 10.17600/18000443).

## Appendix 2

Characteristics of most common genus of phytoplankton, their phytoplankton size range, length, diameter, trophic mode (TM; H: Heterotrophic; M: Mixotrophic; P: Photosynthetic; Pa: Parasitism), dominant pigment (DP; G: Green; YB: Yellow‐brown; NP: non‐pigmented), phytoplankton types (Centric diatoms: CD; Pennate diatoms: PD; Green algae: GA, Dinoflagellates: D; H: Haptophyta; Cr: Cryptophyta; R: Raphidophyceae; Co: Coccolithophore; Cs: Crysophytes), Harmful Algal Bloom (NK: Not known, HNT: Harmful nontoxic; or HAB; Lundholm et al. 2025) and references.

**Table:**

Genus	Species of study	Size range	Length (µm)	Diameter (µm)	TM	DP	Type	HAB	References
Actinocyclus	Actinocyclus sp.	Micro		~30	P	YB	CD	NK	Vidal et al. (2018); Agarwal et al. (2021)
Actinoptychus	*Actinoptychus undulatus*; *Actinoptychus splendens*	Micro		~30	P	YB	CD	NK	Friedrichs et al. (2013); Agarwal et al. (2021)
Akashiwo	Akashiwo sanguinea	Micro	45–65	27.3–40	M		D	HAB	Jeong et al. (2005); Escobar‐Morales and Hernández‐Becerril (2015); García‐Oliva et al. (2022)
Alexandrium	*Alexandrium minutum*; Alexandrium hiranoi; *Alexandrium ostenfeldii*; Alexandrium insuetum; Alexandrium tamutum	Micro		24–31	M		D	HAB	Jeong et al. (2005); Agarwal et al. (2021)
Amphidoma	Amphidoma languida	Nano	10–15	18.4–25.8			D	HAB	Nézan et al. (2012); Wietkamp et al. (2020)
Ansanella	Ansanella granifera	Nano	9.6–15.5	7.3–12.4	M		D	NK	Kang et al. (2023)
Azadinium	Azadinium dexteroporum; Azadinium spinosum; Azadinium trinitatum	Nano	12–16	7‐11			D	HAB	Tillmann et al. (2009); Jauffrais et al. (2012)
Bathycoccus	Bathycoccus prasinos	Pico	1–2	1–2	P	G	GA	NK	Moreau et al. (2012)
Biecheleria	Biecheleria natalensis; Biecheleria cincta; Biecheleria brevisulcata	Micro	9.7–35	8.3–12.2	M	YB	D	NK	Moestrup et al. (2009); Dawut et al. (2018); Sampedro et al. (2022)
Ceratium	Ceratium furcoides; Ceratium sp.	Micro	80–700	~40			D	NK	Masquelier et al. (2011); Agarwal et al. (2021)
Chaetoceros	Chaetoceros tenuissimus; *Chaetoceros affinis*; Chaetoceros sp.; *Chaetoceros curvisetus* 2b; *Chaetoceros curvisetus* 1; *Chaetoceros eibenii*; *Chaetoceros decipiens*; *Chaetoceros socialis*; Chaetoceros neogracilis; *Chaetoceros danicus*; Chaetoceros brevis_2; Chaetoceros protuberans; *Chaetoceros debilis*; *Chaetoceros diadema* 1; *Chaetoceros costatus*; *Chaetoceros didymus* 2; Chaetoceros elegans *Chaetoceros* sp. Clade Na11C3*;* *Chaetoceros constrictus*; *Chaetoceros pseudocurvisetus*; *Chaetoceros anastomosans*; *Chaetoceros lorenzianus*; Chaetoceros sp. Clade Na13C1; *Chaetoceros mitra*; Chaetoceros sporotruncatus; Chaetoceros_lauderi; *Chaetoceros debilis* 1; Chaetoceros contortus cf var contortus; Chaetoceros circinalis; Chaetoceros calcitrans; Chaetoceros cf convolutus	Micro	6–45	4–85	P	YB	CD	HNT	Masquelier et al. (2011); Agarwal et al. (2021)
Chlamydomonas	Chlamydomonas sp.	Nano		10	P	G	GA	NK	Polukhina et al. (2016)
Chloropicon	Chloropicon roscoffensis	Pico	1–3	1–3	P	G	GA	NK	Lopes dos Santos et al. (2017); Ebenezer et al. (2022)
Chrysochromulina	Chrysochromulina sp.; Chrysochromulina campanulifera; Chrysochromulina scutellum; Chrysochromulina rotalis	Nano	3–13		P		H	HAB	Ashour and Sayegh (2021)
Crustomastigaceae‐AB	Crustomastigaceae‐AB sp.	Pico					GA	NK	
Cryptomonadales	Cryptomonadales XX_sp.	Nano	4.5–8.5	3–5	P		Cr	HAB	Lane and Archibald (2008)
Dinophysis	*Dinophysis acuminata*; *Dinophysis norvegica*; *Dinophysis caudata*	Micro	30–100	~20–50			D	HAB	Masquelier et al. (2011); Agarwal et al. (2021); Séchet et al. (2021)
Ditylum	*Ditylum brightwellii*	Micro	40–300	14–120	P	YB	CD	NK	Grattepanche et al. (2011); Agarwal et al. (2021)
Dolichomastigaceae‐B (or Mamiellophyceae)	Dolichomastigaceae‐B sp.	Pico	0.5–7		P	G	GA	NK	Marin and Melkonian (2010); Ribeiro et al. (2024)
Eucampia	Eucampia sp.	Micro	8–100	~20	P	YB	CD	HNT	Masquelier et al. (2011); Agarwal et al. (2021)
Falcomonas	Falcomonas sp.	Nano	10	5			Cr	NK	Atteia et al. (2024)
Fibrocapsa	Fibrocapsa japonica	Micro	20–30	15–20			R	HAB	Vrieling et al. (1995)
Gephyrocapsa	Gephyrocapsa sp.	Nano		~6	P		Co	NK	Agarwal et al. (2021)
Gonyaulax	*Gonyaulax spinifera*; *Gonyaulax verior*	Micro	24–50	30–40	P		D	HAB	Masquelier et al. (2011); Agarwal et al. (2021)
Guinardia	*Guinardia delicatula*; *Guinardia flaccida*; Guinardia striata	Micro	20–250	8–40	P	YB	CD	HNT	Masquelier et al. (2011); Agarwal et al. (2021)
Gymnodinium	Gymnodinium sp.; Gymnodinium litoralis; Gymnodinium nolleri; Gymnodinium aureolum; Gymnodinium microreticulatum; Gymnodinium dorsalisulcum	Micro	40–75	10–34.5			D	HAB	Jeong et al. (2005); Agarwal et al. (2021)
Haptolina	Haptolina sp.; Haptolina hirta	Nano	< 20				H	NK	Piwosz (2019); Mitra et al. (2023)
Heterocapsa	Heterocapsa pygmaea; *Heterocapsa triquetra*; Heterocapsa niei; Heterocapsa nei/rotundata; Heterocapsa sp.; Heterocapsa psammophila; Heterocapsa arctica	Micro	9–45	5–15	M		D	HAB	Uysal et al. (2003); Jeong et al. (2005); Iwataki (2008); Agarwal et al. (2021)
Karlodinium	Karlodinium sp.; Karlodinium veneficum	Nano	8–12				D	HAB	Place et al. (2012); Escobar‐Morales and Hernández‐Becerril (2015)
Lauderia	*Lauderia borealis*	Micro	20–96	15–75	P	YB	CD	NK	Barnett et al. (2024); Agarwal et al. (2021)
Lepidodinium	Lepidodinium sp.; Lepidodinium chlorophorum	Micro	16.8–25	20	M		D	HNT	García‐Oliva et al. (2022); Zhang et al. (2024)
Leptocylindrus	Leptocylindrus aporus; *Leptocylindrus danicus*; *Leptocylindrus minimus*	Micro	20–50	5–16	P	YB	CD	HNT	Masquelier et al. (2011); Agarwal et al. (2021)
Levanderina	Levanderina fissa	Micro	43−72	33.2–54.5			D	HNT	Escobar‐Morales and Hernández‐Becerril (2015); Hernández Márquez et al. (2024)
Mamiella	Mamiella gilva	Pico	< 3	< 3	P	G	GA	NK	Viprey et al. (2008)
Mantoniella	Mantoniella beaufortii; Mantoniella clade A; Mantoniella clade B; Mantoniella antarctica; Mantoniella squamata	Nano		2.8–5.2	P	G	GA	NK	Yau et al. (2020)
Micromonas	Micromonas commode A1; Micromonas commode A2; Micromonas clade B5; *Micromonas pusilla*; Micromonas bravo B1; Micromonas bravo B2; Micromonas polaris	Pico	1–2		P	G	GA	NK	Worden et al. (2009); Moreau et al. (2012)
Minidiscus	Minidiscus trioculatus	Nano		2.5–6.8	P	YB	CD	NK	Quiroga and Chrétiennot‐Dinet (2004); Li et al. (2020)
MOCH‐2	MOCH‐2 XXX sp.	Pico		< 3			MOCH	NK	Giner et al. (2019)
Navicula	Navicula perminuta; *Navicula phyllepta*; Navicula sp.	Micro		~5.6–16	P	YB	PD	NK	Agarwal et al. (2021)
Ostreococcus	Ostreococcus tauri; Ostreococcus clade B; Ostreococcus lucimarinus	Pico	0.8–2		P	G	GA	NK	Courties et al. (1994); Moreau et al. (2012)
Parmales_env_2	Parmales env 2 X sp.	Pico		2‐5			Cs	NK	Ichinomiya et al. (2016); Kuwata et al. (2018)
Parmales_env_3 A	Parmales env 3A sp.	Pico		2–5			Cs	NK	Ichinomiya et al. (2016); Kuwata et al. (2018)
Phaeocystis	*Phaeocystis globosa*; Phaeocystis sp.; *Phaeocystis pouchetii*; Phaeocystis jahnii; Phaeocystis cordata; Phaeocystis rex	Nano	4‐6	2.9–100	P		H	HAB	Jakobsen and Tang (2002); Rousseau et al. (2013); Agarwal et al. (2021)
Picochlorum	Picochlorum sp.	Pico	1–4	1–4	P	G	GA	NK	Goswami et al. (2022); Ma et al. (2021)
Plagiogrammopsis	Plagiogrammopsis vanheurckii	Nano	18	3.5	P	YB	CD	NK	Garcia (2016)
Pleurosigma	Pleurosigma sp.	Micro	90–220	26–50	P	YB	PD	NK	Masquelier et al. (2011); Agarwal et al. (2021)
Polar‐centric Mediophyceae	Polar‐centric*‐Mediophyceae* X sp.	Micro			P	YB	CD	NK	Davidovich et al. (2017)
Prasino‐Clade‐VIII	*Prasino*‐Clade‐VIII XXX sp.	Pico			P	G	GA	NK	Not et al. (2004); Viprey et al. (2008)
Prasinoderma	Prasinoderma sp.; Prasinoderma coloniale; Prasinoderma singularis	Pico		3–8	P	G	GA	NK	Not et al. (2004)
Proboscia	Proboscia sp.	Nano		2.5–20	P	YB	PD	NK	Van De Meene and Pickett‐Heaps (2002); Masquelier et al. (2011)
Prorocentrum	*Prorocentrum triestinum*; Prorocentrum sp.; Prorocentrum donghaiense; Prorocentrum rhathymum; *Prorocentrum mexicanum*; Prorocentrum texanum	Micro	20–50	6–29			D	HAB	Jeong et al. (2005); Masquelier et al. (2011); Agarwal et al. (2021)
Protoceratium	Protoceratium reticulatum	Micro	27	25			D	HAB	Erga et al. (2015)
Prymnesiophyceae Clade_B3	Prymnesiophyceae Clade B3 X sp.	Nano					Co, H	NK	Edvardsen et al. (2011, 2016); Gran‐Stadniczeñko et al. (2017)
Prymnesiophyceae Clade_B4	Prymnesiophyceae Clade B4 X sp.	Nano					Co, H	NK	Edvardsen et al. (2011, 2016); Gran‐Stadniczeñko et al. (2017)
Prymnesiophyceae Clade_D	Prymnesiophyceae Clade D XX sp.	Nano					Co, H	NK	Edvardsen et al. (2011, 2016); Gran‐Stadniczeñko et al. (2017)
Prymnesiophyceae Clade_F	Prymnesiophyceae Clade F XX sp.	Nano					Co, H	NK	Edvardsen et al. (2011, 2016); Gran‐Stadniczeñko et al. (2017)
Prymnesium	Prymnesium sp.; Prymnesium minus; Prymnesium kappa; Prymnesium palpebrale; Prymnesium zebrinum; Prymnesium radiatum; Prymnesium polylepis; Prymnesium aff polylepis	Nano		7–9			H	HAB	Granéli and Johansson (2003)
Pseudohaptolina	Pseudohaptolina birgeri	Nano		6–14			H	NK	Edvardsen et al. (2011)
Pseudo‐nitzschia	Pseudo‐nitzschia granii	Micro	25–160		P	YB	PD	HAB	Agarwal et al. (2021)
Pycnococcus	Pycnococcus provasolii	Pico	1.5–4	1.5–4	P	G	GA	NK	Vaulot et al. (2008)
Pyramimonadales	Pyramimonadales XXX sp.	Nano	6–9.5	5–7.2	P	G	GA	NK	Daugbjerg et al. (2020); Haraguchi et al. (2022)
Pyramimonas	Pyramimonas sp.; Pyramimonas australis; Pyramimonas aurea; Pyramimonas parkeae; Pyramimonas vacuolata	Nano		~8	P	G	GA	NK	Agarwal et al. (2021)
Radial‐centric‐basal Coscinodiscophyceae	Radial‐centric‐basal‐*Coscinodiscophyceae* X sp.	Micro	30–500		P	YB	CD	NK	
Scrippsiella	Scrippsiella sp.; Scrippsiella acuminata; Scrippsiella precaria	Micro	20–30	16–26			D	HNT	Jeong et al. (2005); Masquelier et al. (2011); Agarwal et al. (2021)
Suessiales	Suessiales XX sp.	Micro	8.8–11.4	6.0–7.5			D	NK	Siano et al. (2010)
Teleaulax	Teleaulax acuta; Teleaulax sp.	Nano	5–18	4.1–7.1	P		Cr	NK	Lee et al. (2014)
Tetraselmis	Tetraselmis sp.	Nano	2–20		P	G	GA	NK	Guillou et al. (2004)
Thalassiosira	Thalassiosira oceanica; *Thalassiosira minima*; *Thalassiosira pseudonana*; Thalassiosira curviseriata; *Thalassiosira delicatula*; Thalassiosira sp.	Micro	7–56	2–40	P	YB	CD	NK/HNT	Agarwal et al. (2021)
Tripos	Tripos fusus; Tripos longipes; Tripos furca	Micro	300–600	25–27			D	HNT	Hallegraeff et al. (2020)
Woloszynskia	Woloszynskia sp.; Woloszynskia halophila	Micro	17–35	12–32			D	HAB	Kremp et al. (2005)
